# Assessing SABU (Serum Anti Bisa Ular), the sole Indonesian antivenom: A proteomic analysis and neutralization efficacy study

**DOI:** 10.1038/srep37299

**Published:** 2016-11-21

**Authors:** Choo Hock Tan, Jia Lee Liew, Kae Yi Tan, Nget Hong Tan

**Affiliations:** 1 Department of Pharmacology, Faculty of Medicine, University of Malaya, 50603 Kuala Lumpur, Malaysia; 2 Department of Molecular Medicine, Faculty of Medicine, University of Malaya, 50603 Kuala Lumpur, Malaysia

## Abstract

Serum Anti Ular Bisa (SABU) is the only snake antivenom produced locally in Indonesia; however, its effectiveness has not been rigorously evaluated. This study aimed to assess the protein composition and neutralization efficacy of SABU. SDS polyacrylamide gel electrophoresis, size-exclusion liquid chromatography and shotgun proteomics revealed that SABU consists of F(ab’)_2_ but a significant amount of dimers, protein aggregates and contaminant albumins. SABU moderately neutralized *Calloselasma rhodostoma* venom (potency of 12.7 mg venom neutralized per ml antivenom, or 121.8 mg venom per g antivenom protein) and *Bungarus fasciatus* venom (0.9 mg/ml; 8.5 mg/g) but it was weak against the venoms of *Naja sputatrix* (0.3 mg/ml; 2.9 mg/g), *Naja sumatrana* (0.2 mg/ml; 1.8 mg/g) and *Bungarus candidus* (0.1 mg/ml; 1.0 mg/g). In comparison, NPAV, the Thai Neuro Polyvalent Antivenom, outperformed SABU with greater potencies against the venoms of *N. sputatrix* (0.6 mg/ml; 8.3 mg/g), *N. sumatrana* (0.5 mg/ml; 7.1 mg/g) and *B. candidus* (1.7 mg/ml; 23.2 mg/g). The inferior efficacy of SABU implies that a large antivenom dose is required clinically for effective treatment. Besides, the antivenom contains numerous impurities e.g., albumins that greatly increase the risk of hypersensitivity. Together, the findings indicate that the production of SABU warrants further improvement.

Indonesia is a vast archipelago extending more than 5000 km from east to west in the equatorial region. Its rich herpetofauna includes more than 10 venomous snake species that distribute in two major ecozones divided by the Wallace’s line. On the eastern side of the Wallace’s line on the Sahul Shelf, there are the Australian elapid fauna, while snakes inhabiting islands west of the Wallace’s line on the Sunda Shelf are mostly common or similar species found in the Malay Archipelago. Java and Sumatra are two huge, densely populated islands on the Sunda Shelf, and they are also natural habitat to many Indonesian snakes. In these islands, the spitting cobras (*Naja sputatrix* in Java and Lesser Sunda*; Naja sumatrana* in Sumatra and Kalimantan), the Malayan krait (*Bungarus candidus*) (Sumatra and Java) and the Malayan pit viper (*Calloselasma rhodostoma* in Java) are listed under WHO Category 1 of medical importance^1^. Other species of medical importance include the Russell’s viper (*Daboia siamensis*) and green pit vipers of *Trimeresurus* complex, the geographical distributions of which are relatively limited in the country. Although snakebite is likely affecting the Indonesian population at a large scale^1^, unfortunately, comprehensive epidemiological study of snakebite in this country remains extremely scarce^2^.

Snakebite envenomation has been aptly described as a disease of poverty that affects heavily the poor or rural population in the developing tropical countries^3^,^4^. Prior to the year 2015, it was obscurely listed under “Other Categories” of the Neglected Tropical Diseases by the WHO, lacking systematic attention and official global support program. In 2015, the world saw the de-listing of this critical health problem from the mentioned list of WHO Neglected Tropical Diseases. In fact, the persistent underestimation of snakebite morbidity and mortality has made it the most neglected condition among many other diseases in the tropics^5^, and toxinology experts have called on WHO and governments to re-establish snakebite as a neglected tropical disease^6^. Regional toxinologists are also taking up proactive approaches to tackle the various challenges associated with snakebite envenomation. One of the basic steps to overcome the problem is to have a rigorous assessment of antivenom in order to ensure the supply of an affordable and efficacious antivenom product^7^. Various techniques have been adopted for antivenom assessment, including the use of high performance liquid chromatography to profile antivenom proteins^8^, and enzyme-linked immunosorbent assay as well as affinity chromatography (antivenomic approach) to characterize the immunological binding between antivenom and toxins^7^,^9^. Nevertheless, *in vivo* study remains indispensable to determine the efficacy of an antivenom in neutralizing the overall toxic effect of snake venom. The measurable dose-response data obtained from *in vivo* study will provide an objective reference for the comparison of efficacy between different antivenom products^5^,^10^,^11^.

In Indonesia, the only local antivenom available is marketed as Biosave^®^, which is more commonly known as SABU (Serum Anti Bisa Ular), manufactured by the state-owned enterprise BioFarma. SABU is formulated as a trispecific or trivalent antivenom for clinical use in Indonesia (except the region east of the Wallace’s line and West Papua). It is derived from the sera of horses which have been hyperimmunized against the venoms from three snake species of Indonesian origin: the Javan spitting cobra (*Naja sputatrix*, ular sendok Jawa), the Malayan pit viper (*Calloselasma rhodostoma*, ular tanah) and the banded krait (*Bungarus fasciatus*, ular welang). This is an antivenom packaged in liquid form, demanding cold-chain transport and stringent storage condition maintained between 2–8 °C. Anecdotally, SABU is not widely available in many regions of the country, and reports of the use and efficacy of SABU have been lacking. The effectiveness and limitation of this antivenom have not been rigorously evaluated, leaving the manufacturer and healthcare community clueless about its usefulness and weakness in snakebite envenomation treatment. In this study, we investigated the quality of SABU including analysis of its protein composition and neutralization capacity against the toxic effects induced by the venoms of important snakes in Indonesia. Parallel to this, the performance of SABU was compared to two other antivenom products available commercially in Southeast Asia, i.e. Neuro Polyvalent Antivenom (NPAV) and Hemato Polyvalent Antivenom (HPAV) which are produced by The Thai Red Cross Society, Queen Saovabha Memorial Institute, Bangkok. It is hoped that the findings will provide insights into the strength and weakness of the Indonesian antivenom, and shed light on how the production and the use of the antivenom can be optimized.

## Results

### Protein determination

SABU has a protein concentration of 104.3 ± 0.5 mg/ml (of undiluted liquid antivenom, 5 ml), equivalent to approximately 520 mg protein per vial of antivenom. NPAV, in comparison, has a protein concentration of 75.3 ± 0.6 mg/ml (of reconstituted antivenom, 10 ml), equivalent to approximately 750 mg protein per vial of antivenom. HPAV has a protein concentration of 43.0 ± 0.5 mg/ml (of reconstituted antivenom, 10 ml), equivalent to approximately 430 mg protein per vial of antivenom.

### Electrophoretic and chromatographic profiling of antivenom

**Figure 1:**
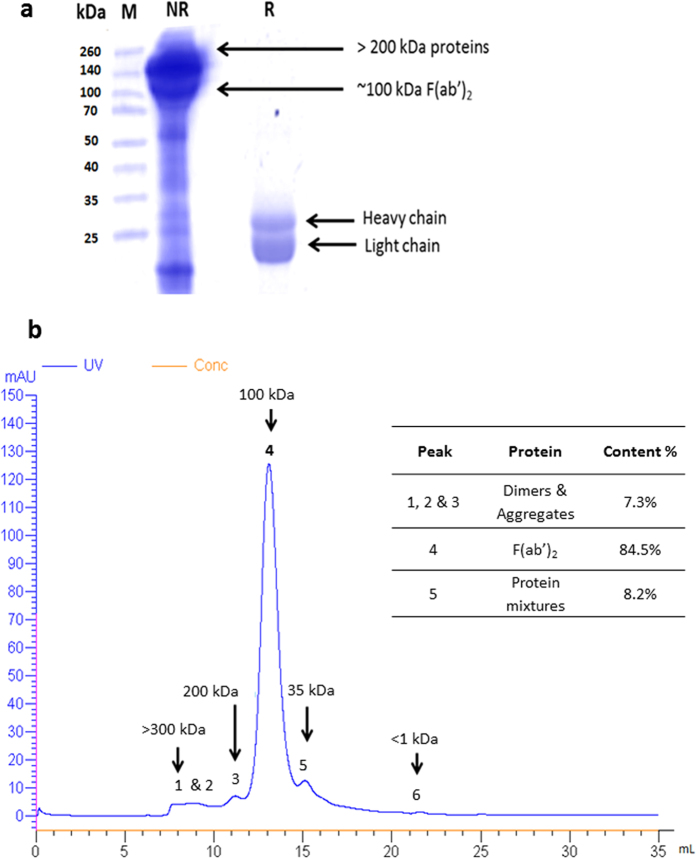
Profiling of Serum Anti Bisa Ular (SABU), the Indonesian tri-specific antivenom. (**a**) SDS-PAGE of SABU under non-reducing (NR) and reducing (R) conditions. M: Molecular mass standard. (**b**) Size-exclusion FPLC of SABU (flow rate = 0.5 ml/min). Inlet shows the protein abundance estimated by peak areas under the curve.

The electrophoretic profile of SABU was shown in Fig. 1a. Non-reducing SDS-PAGE of SABU revealed the presence of major proteins with molecular mass above 100 kDa. On reducing SDS-PAGE, these proteins were observed mainly as two major bands at 21 and 25 kDa. Size-exclusion (or gel filtration) fast protein liquid chromatography (FPLC) resolved the antivenom into 5 peaks corresponding to the elution of proteins of different molecular masses (Fig. 1b). The major proteins were eluted in Peak 4, estimated to constitute 84.5% of total antivenom proteins based on the peak area under the curve. The calibrated estimated molecular mass for peak 4 proteins is approximately 100–110 kDa. SABU was also found to contain a significant amount of proteins above 200 kDa (Peaks 1–3) (7.3%) and in the range of 30–60 kDa, detected in Peak 5 (8.2%) (Fig. 1b).

### Proteomic analysis with liquid chromatography-tandem mass spectrometry (LC-MS/MS)

**Figure 2:**
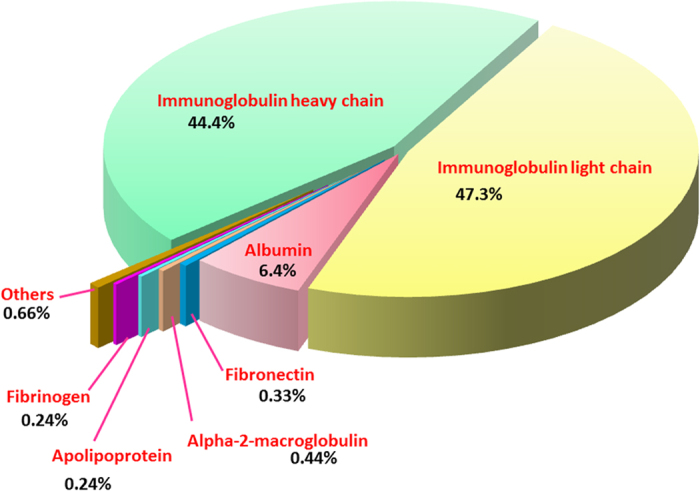
Proteome of the Indonesian tri-specific antivenom, Serum Anti Bisa Ular (SABU).

The shotgun LC-MS/MS analysis revealed the protein composition of fractions collected from the size-exclusion FPLC (Table 1; Fig. 1b). The protein scores, mass spectral data (intensities, masses and charges of ions) and amino acid sequences were provided in supplementary file Table S1. Collectively, immunoglobulin chains were detected as the main composition throughout Peak 1 to Peak 3, constituting 5.73% of total antivenom proteins. These peaks represented high molecular mass proteins (>200 kDa). Peak 4 (corresponding to the molecular mass range of 100–120 kDa) also contained immunoglobulin chains as its main component, and these immunoglobulin chains accounted for 84.04% of total antivenom proteins. The immunoglobulin chains were composed of heavy chains and light chains in a ratio of approximately 1:1. Peak 5, with molecular mass of 30–60 kDa, consisted mainly of equine serum albumins (two isoforms were identified) as well as some fragments of serum proteins such as immunoglobulin heavy/light chains, serum fibronectin and serotransferrin. Peak 5 constituted 8.2% of total antivenom proteins, serum albumins, contributed to approximately 5.5% of the total antivenom proteins in SABU. The overall protein composition of SABU is shown in Fig. 2.

### Immunological binding of venom antigens

**Figure 3:**
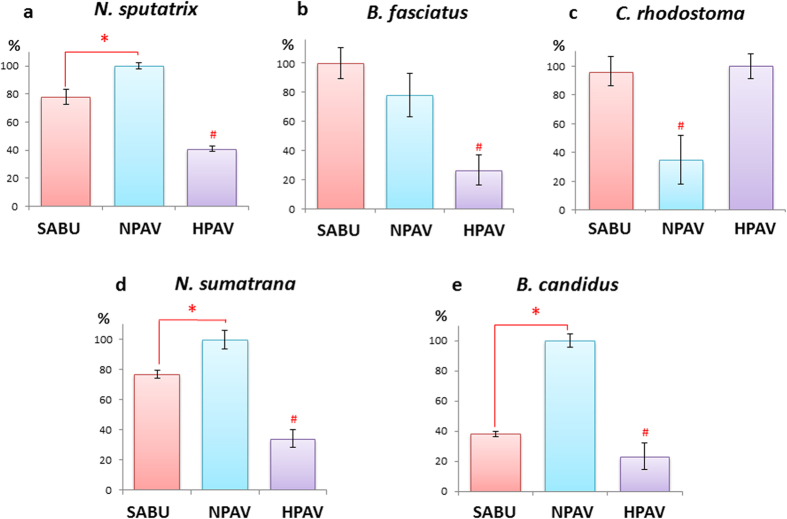
Immunological binding activities of antivenoms toward (**a**) *Naja sputatrix*; (**b**) *Bungarus fasciatus*; (**c**) *Calloselasma rhodostoma*; (**d**) *Naja sumatrana*; (**e**) *Bungarus candidus*. Values were percentages of normalized mean absorbance ± S.E.M. *Significant difference, *p* < 0.05; # Non-specific negative control.

The immunological binding activities of SABU toward the antigens of the five venoms tested were shown in Fig. 3. Compared with NPAV (binding activity = 100%), SABU was found to be significantly weaker in binding the venom antigens of *N. sputatrix*, *N. sumatrana* and *B. candidus* (*p* < 0.05). The binding activity of SABU toward *B. candidus* venom antigens was in particular the weakest (38.0 ± 1.9%), although its binding toward the venom antigens of another krait species, *B. fasciatus*, was comparable to that of NPAV (*p* > 0.05). Against *C. rhodsotoma* venom antigens, SABU was shown to be as effective as HPAV in immunological binding (95–100%). In assays that tested antivenom binding of elapid venoms of cobras and kraits, HPAV served as the negative control. On the other hand, NPAV served as the negative control in assay that tested antivenom binding of *C. rhodostoma* venom.

### Neutralization of procoagulant (thrombin-like) activity of *C. rhodostoma* venom

The minimal coagulant dose (MCD) of *C. rhodostoma* venom on bovine fibrinogen was determined to be 0.53 ± 0.06 μg. Both SABU and HPAV were equally effective to neutralize the procoagulant effect of the venom at 2 MCD. The effective dose that prolonged the onset of clotting to 3 times of that induced by 2 MCD was determined to be 0.4 ± 0.0 μl antivenom, or 2.65 mg venom per ml of the antivenom (SABU and HPAV). For consistency, the effective doses were normalized by the protein contents of the respective antivenoms. The normalized effective doses are25 mg venom per gram protein for SABUand 62 mg venom per gram protein for HPAV, respectively.

### Lethality of venom and neutralization by antivenom

Table 2 shows the intravenous median lethal doses (LD_50_) of the venoms and the efficacy of different antivenoms in neutralizing the lethal effect of the venoms. For comparison purpose, the potency of antivenom neutralization was expressed in mg/ml (amount of venom neutralized per milliliter antivenom) and mg/g (amount of venom neutralized per gram protein of antivenom).

## Discussion

Most antivenoms available clinically in Asia are formulated as F(ab’)_2_,. The pharmacokinetics of F(ab)’_2_ varies from that of whole IgG antivenom although the efficacy may not differ markedly, for instance, between an Australian F(ab’)_2_ antivenom and a new IgG antivenom used in treating *Oxyranus scutellatus* envenoming in Papua New Guinea^12^. In this study, the active biologic of SABU is determined to be the dimeric F(ab’)_2_ with a molecular mass of 100–120 kDa. Each Fab monomer is made up of a cleaved heavy chain (25 kDa) and a light chain (21 kDa), consistent with the electrophoretic profile under reducing condition^8^. This is also supported by the size-exclusion FPLC of SABU, where the elution time and the calibrated molecular mass of the major peak is consistent with that of F(ab’)_2_ reported previously^8^. Importantly, in this study, the presence of the heavy and light chains of immunoglobulins in the major peak was validated through a proteomic approach using LCMS/MS. F(ab’)_2_ is a pepsin-digested product of immunoglobulin G; it exists as a bivalent molecule of two covalently bonded antigen-binding fragments (Fab), each of which is composed of a heavy chain and a light chain. The Fab molecule carries the paratopes for antigen binding^13^. Currently, this is the main form of antivenom immunoglobulin produced by most antivenom manufacturers, preferred over the IgG form which contains the allergenic Fc fragment^14^. F(ab)’_2_ antivenoms generally have a long elimination half-life (up to 72 hours) and a reasonably good volume of distribution, thereby reducing the likelihood of venom-antivenom mismatch and recurrent syndrome (which is more common with monomeric Fab preparation) when given in adequate doses^13^,^15^. In addition, F(ab’)_2_ is large enough to be excluded from glomerular filtration, hence they do not cause obstructive tubulopathy as seen with some Fab preparations^13^.

The presence of immunoglobulin chains and some minor serum proteins e.g., macroglobulin in Peaks 1–3 (>200 kDa, Fig. 1b) indicated the presence of dimers and protein aggregates in SABU. These are proteins that can increase the risk of pyrogenic and hypersensitive reactions in patients treated with antivenom^16^. Protein aggregation in immunoglobulin products had been shown to be associated with phenol^17^, a commonly used preservative in antivenoms including the liquid product SABU. Besides, the commonly used ammonium sulfate precipitation method to purify IgG/F(ab’)_2_ is also known to induce aggregate formation^14^. In comparison, NPAV, the Thai antivenom with F(ab’)_2_ formulation, consisted of negligible amount (< 0.1%) of the high molecular mass proteins (Leong *et al*.^18^). The difference could be due to the different purification method (caprylic acid precipitation) adopted by the Thai producer^19^, which allows for the production of antivenoms of higher purity and a lower aggregate content^14^. Clinical studies have also shown that the incidence of early reactions to Thai antivenoms (manufactured by Queen Saovabha Memorial Institute) was low^20^. Unfortunately, the data of hypersensitivity associated with SABU has not been available in published literature. In addition, compared to the size-exclusion FPLC profiles of the Thai polyvalent antivenoms^8^,^18^, SABU showed an additional chromatographic peak in the range of 30–60 kDa molecular mass (Peak 5, Fig. 1b), which contained predominantly equine serum albumins. The presence of contaminant i.e. serum albumins in SABU was probably due to inadequate purification process to separate IgG from other plasma proteins at the early stage of antivenom production. Equine serum albumin is a known causative factor of hypersensitivity to horse serum-based antivenom, which can be life-threatening as patients may develop respiratory distress and shock from anaphylactoid reaction^14^,^21^. The finding hence indicates the need for more stringent quality control of SABU purity, where the albumin content should ideally not exceed 1% of the total proteins in accordance with the WHO guideline on antivenom production^14^. Clinically, despite the lack of data on adverse reactions associated with SABU, it is expected that patients receiving SABU treatment are at higher risk of developing hypersensitive reaction. Therefore, proper resuscitation facility and close monitoring of the patient before and during SABU treatment are warranted.

In this study, the venoms of *N. sputatrix*, *B. fasciatus* and *C. rhodostoma* of Indonesian origin (used as the immunogen in the production of SABU) were considered as homologous to the antivenom SABU. *Calloselasma rhodostoma* is a monotypic terrestrial pit viper; in Indonesia its distribution is mainly restricted to the eastern Java^22^. Its venom is known to be procoagulant and hemotoxic, but distinct from many other arboreal pit vipers of the *Trimeresurus* complex in terms of toxin antigenicity and neutralization profile^23^,^24^,^25^. On the other hand, cobras (*Naja* sp.) and kraits (*Bungarus* sp.) in Indonesia consist of two to three distinct species; bites by these elapids can cause rapid neuromuscular paralysis, respiratory failure and death within minutes to hours^1^,^10^. In this study, venoms of the other medically important cobra (*N. sumatrana*) and krait (*B. candidus*) were also included to examine the cross-reactivity and cross-neutralization capacity of SABU against them. Notably, the intravenous median lethal doses (LD_50_) of the venoms of the two spitting cobras vary in mice: *N. sumatrana* venom is twice more lethal than *N. sputatrix* venom. This is in agreement with the higher content of alpha-neurotoxins in *N. sumatrana* venom compared to *N. sputatrix* venom, which has a much higher content of cytotoxins^26^. The LD_50_ value of cobra cytotoxin is about 10-fold higher (hence less lethal) than the alpha-neurotoxin when administered intravenously^9^,^27^,^28^. On the other hand, krait venoms are known to exhibit high lethality (LD_50_ < 0.2 μg/g, including Indonesian *B. candidus*), except for *B. fasciatus* of Thailand which has an exceptionally higher venom LD_50_ exceeding 1.0 μg/g^18^,^29^. The Indonesian *B. fasciatus*, interestingly, has a much lower venom LD_50_ (0.45 μg/g) than the Thai species. The phenomenon indicates intra-specific venom variation which could be due to geographical factor, as demonstrated previously for the Malaysian and Indonesian king cobras^30^. Remarkable geographical venom variations had also been reported within the species of several Asiatic cobras^31^,^32^,^33^,^34^, and the biological significance and medical ramification of these variations cannot be overlooked. Differences in the venom profiles of the Thai and Indonesian *B. fasciatus* deserve further investigation.

The use of ELISA enables the comparison of immunological binding activities of different antivenoms toward the antigens of a specific venom. The assay provides quick “screening” of the immunoreactivity and immunorecognition of an antivenom against venom antigens, although the result (positive finding) can sometimes be misleading as it is not necessarily congruent with *in vitro* and *in vivo* neutralization effectiveness^9^, as immunological binding also involves venom proteins which do not contribute to toxicity. In this study, the immunological binding assay showed that both SABU and HPAV bound equally well to *C. rhodostoma* venom antigens. However, the ELISA result differed to some extent from the neutralization of the procoagulant and lethal effects of *C. rhodostoma* venom, where the Thai antivenom (Hemato Polyvalent Antivenom) was found to be more superior when comparing the potency values of the two antivenoms in mg venom per g antivenom protein. When SABU and NPAV were challenged against *N. sputatrix* venom at 5 LD_50_ challenge dose, SABU failed to neutralize the lethal effect (with zero survival rate), whereas NPAV only protected 25% of envenomed mice (at the maximum permissible antivenom volume for intravenous injection, 200 μl). At a lower challenge dose (2.5 LD_50_), both antivenoms neutralized the venom to varying degrees: SABU weakly neutralized the venom with a potency of 0.30 mg/ml or 2.9 mg/g, while NPAV, despite being a heterologous antivenom, was able to cross-neutralize the venom at a much greater potency (0.62 mg/ml or 8.3 mg/g), which was approximately 3-fold higher than SABU. This information is clinically useful as it indicates that the Javan spitting cobra *N. sputatrix* and the Thai monocled cobra (*Naja kaouthia*) share toxin epitopes that can be neutralized by SABU or NPAV, although the potency or efficacy of SABU apparently requires further optimization. When tested against the heterologous *N. sumatrana* venom, NPAV was also noted to outperform SABU in neutralizing the lethal effect of the venom by a 4-fold greater potency. These *in vivo* neutralization findings were supported by the immunological binding results, where the immunoreactivity of SABU against both cobra venoms was significantly lower than that of NPAV. From the practical standpoint, the consistently low potency of SABU (0.2–0.3 mg/ml) against the two Indonesian cobra venoms implies that each vial of SABU (5 ml) may only neutralize 1–1.5 mg venom. Clinically, a higher dosage of SABU, perhaps in multiple repeated doses, is likely needed for effective neutralization considering that cobras are capable of injecting huge amount of venom (50–100 mg or above for an adult snake, based on the author’s milking experience). In Thailand, the initial dose of treating cobra envenomation has been recommended at 10 vials of antivenom, with additional doses as required based on clinical judgment. Surprisingly, despite its lower neutralization potency, the initial dose of SABU has been recommended at 2 vials, with 1 additional vial given after 6 hours if required^2^. In comparison with the Thai antivenom, 2 vials of SABU is likely under-dosed and the unfavorable clinical implication is evident. Hence, proper measures need to be taken to ensure adequate dosing for the treatment of snakebite envenomation in the country.

When tested against the krait venoms, the neutralization of Indonesian *B. fasciatus* venom was only slightly weaker for SABU compared with NPAV (SABU, P = 8.5 mg/g; NPAV, P = 11.5 mg/g), in agreement with the ELISA finding where no significant difference was noted between the immunoreactivity of the two antivenoms. However, SABU neutralization against the heterologous Malayan krait (*B. candidus*) venom (Indonesian origin) is extremely poor (P = 0.10 mg/ml or 0.9 mg/g), implying that the antigenicity of *B. fasciatus* and *B. candidus* venoms varies remarkably. This is supported by the fact that in Thailand, envenomation by *B. candidus* or *B. fasciatus* requires the species-specific monovalent antivenom treatment, or the polyvalent antivenom (NPAV) which immunogen includes venoms from both species. Indeed, compared with SABU, NPAV was much more effective in immunological binding and neutralization of the Indonesian *B. candidus* (with a 23-fold greater potency). Therefore, the effectiveness of SABU (raised against *B. fasciatus* venom) in treating *B. candidus* envenomation in Indonesia is likely to be very limited. In fact, *B. candidus* is more frequently encountered and involved in envenomation compared with *B. fasciatus* in Southeast Asia, and deaths resulted from *B. candidus* bite in Indonesia had been reported previously^1^,^2^. An antivenom product that is effective against both krait species (particularly *B. candidus*) is therefore warranted in the country.

## Conclusions

Coupling SDS-PAGE, size-exclusion FPLC and LCMS/MS, this study unveiled the proteome of the Indonesian antivenom SABU, verifying that it is an F(ab’)_2_ antivenom product that also contains a significant amount of protein aggregates and contaminant albumins. The presence of substantial amount of protein aggregates and albumins is of serious safety concern as these components are known risk factors of hypersensitive reaction. Comparing with NPAV (a Thai polyvalent antivenom), the neutralization efficacy of SABU is generally lower even when tested against its homologous venoms of Indonesian origin, despite the higher content of antivenom proteins. The neutralization results reported were based on *in vivo* animal model and *in vitro* assays and hence need to be clinically verified; however, the findings are largely consistent with sporadic reports of low effectiveness of SABU in Indonesia. The inadequate efficacy of SABU could be due to the presence of other “junk proteins” and non-specific F(ab’)_2_ that do not react with the toxin antigens concerned. On the other hand, while antivenom neutralization against cobra and krait toxins (especially the low molecular mass alpha-neurotoxins) is known to be limited^9^,^28^, such “weakness” of antivenom is in particular serious for SABU, judging from its feeble neutralization against the venoms of *N. sputatrix* and *B. candidus*, two medically important species in Java - the most densely inhabited island of Indonesia. From the practical standpoint, the findings indicate the urgency for a revised manufacturing process to ensure the production of a safe and effective antivenom in the country, where it should be furnished with the qualities of improved purity, higher potency and a wider coverage of species in Indonesia.

## Materials and Methods

### Venoms and antivenoms

The venoms of *Naja sputatrix* (Java) and *Calloselasma rhodostoma* (Java) venoms were supplied by Latoxan, France. *Bungarus candidus* (Java) was purchased from Venom Supplies, Australia. *Naja sumatrana* (Sumatra) and *Bungarus fasciatus* (Java) venoms were obtained from local supplier in Indonesia. Venoms were pooled from a minimum of 2 (*Naja sumatrana*) to > 5 adult specimens, and stored as lyophilized samples at −20 °C until use.

Three polyvalent antivenoms were used in this study: (a) Serum Anti Bisa Ular (SABU; batch no. 4701314; expiry date: October, 2016; manufactured by BioFarma Pharmaceuticals, Bandung, Indonesia), a liquid form product with an unknown form of its active molecule. This antivenom was obtained from the sera of horses hyperimmunized against three venoms of Indonesian origin: *Naja sputatrix* (Javan spitting cobra), *Bungarus fasciatus* (banded krait) and *Calloselasma rhodostoma* (Malayan pit viper); (b) Neuro Polyvalent Snake Antivenom (NPAV; batch no. NP00414; expiry date: December 9^th^, 2019), a lyophilized product containing purified F(ab)’_2_ derived from the sera of horses hyperimmunized against the venoms of *Naja kaouthia* (monocled cobra), *Ophiophagus hannah* (king cobra), *Bungarus candidus* (Malayan krait) and *Bungarus fasciatus* (banded krait), all of Thai origin; (c) Hemato Polyvalent Snake Antivenom (HPAV, ; batch no. HP00216; expiry date: March 8^th^, 2021), a lyophilized product containing purified F(ab)’_2_ obtained from the sera of horses hyperimmunized against the venoms of *Calloselasma rhodostoma* (Malayan pit viper), *Cryptelytrops albolabris* (white-lipped pit viper) and *Daboia siamensis* (Russell’s viper), all of Thai origin. Both HPAV and NPAV were manufactured by Queen Saovabha Memorial Institute, Bangkok, Thailand, and were used in the immunological binding and neutralization assays for comparison purpose in this study.

### Animals

Albino ICR Mice (20–25 g) were supplied by the Laboratory Animal Centre, Faculty of Medicine, University of Malaya. The protocol of animal studies was based on the Council for International Organizations of Medical Sciences (CIOMS) guidelines on animal experimentation and was approved by the Institutional Animal Care and Use Committee of the University of Malaya (Ethics clearance number: 2014–09–11/PHAR/R/TCH).

### Materials

Ammonium bicarbonate, dithiothreitol (DTT) and iodoacetamide were purchased from Sigma-Aldrich (USA). Mass-spectrometry grade trypsin protease, Spectra™ Multicolor Broad Range Protein Ladder (10 to 260 kDa), and HPLC grade solvents used in the studies were purchased from Thermo Scientific™ Pierce™ (USA). Millipore ZipTip^®^ C_18_ Pipette Tips were obtained from Merck, USA. Other chemicals and reagents of analytical grade were purchased from Sigma-Aldrich (USA).

### Estimation of antivenom protein concentration

Protein concentrations in antivenoms (SABU, NPAV and HPAV) were determined using Thermo Scientific^TM^ Pierce^TM^ BCA (bicinchoninic acid) Protein Assay Kit. The protein concentrations were expressed as means ± S.E.M. of triplicates.

### Electrophoretic and chromatographic profiling of SABU antivenom

Sodium dodecyl sulfate polyacrylamide gel electrophoresis (SDS-PAGE) of SABU was conducted under reducing and non-reducing conditions using the MiniPROTEAN II Electrophoresis Cell (BioRad, USA) as adapted from Studier^34^. Bio-Rad broad-range prestained SDS-PAGE standards (6.5–200 kDa) and 15 μL SABU (3 mg/ml) were loaded onto 12.5% gel and the electroporesis running condition was standardized as per previously reported^35^. Fast protein liquid chromatography (FPLC) of the antivenom (approximated to 1 mg in 100 μL injection volume) was performed using a Superdex 200 HR 10/30, 13 μm SEC 10 × 300 mm (GE Healthcare, Sweden). Elution buffer was 100 mM sodium phosphate, 0.15 M NaCl, pH 7.4 at a flow rate of 0.5 ml/min. Proteins in the antivenom were detected by absorbance readings at 280 nm for 30 min. The column was calibrated using the following protein standards supplied by Bio-Rad (Bio-Rad Gel filtration Standard): thyroglobulin (670 kDa), α-globulin (158 kDa), ovalbumin (44 kDa) and myoglobin (17 kDa).

### Profiling of proteins using liquid chromatography-tandem mass spectrometry

Proteins in the chromatographic fractions were subjected to reduction with DTT, alkylation with iodoacetamide, and in-solution digestion with mass-spectrometry grade trypsin protease according to the manufacturer’s protocol (Thermo Scientific™ Pierce™, Rockford, IL, USA). The trypsin-digested peptides were desalted with Millipore ZipTip^®^ C_18_ Pipette Tips (Merck, USA) according to the manufacturer’s protocol to enhance the performance of mass spectrometry. The digested peptides (0.5 μL) and 0.5 μL α-cyano-4-hydroxycinnamic acid matrix were mixed and spotted on OPTI-TOF™ LC/MALDI insert plate (123 mm × 81 mm). MALDI-TOF/TOF was performed using AB SCIEX 5800 Plus Analyzer equipped with a neodymium: yttrium-aluminium-garnet laser (laser wavelength was 355 nm). The TOF/TOF calibration mixtures (AB SCIEX, USA) were used to calibrate the spectrum to a mass tolerance within 10 ppm. For MS mode, peptides mass maps were acquired in positive reflection mode and 800–4000 m/z mass range were used with 100 laser shots per spectrum. The MS/MS peak detection criteria used were a minimum signal-to-noise (S/N) of 100. The raw mass spectra acquired were exported to AB SCIEX ProteinPilot™ Software search against all non-redundant NCBI Serpentes database (taxid: 8570, Serpentes). MS peak filter mass range 800–4000 m/z was applied. Precursor and fragment mass tolerances were set to 100 ppm and 0.2 Da respectively and allowing one missed cleavage. Oxidation (M) was set as a variable modification and carbamidomethylation (C) was set as a fixed modification. The protein score intervals (C.I.) above 95% were considered as confident identification. The peptide finger mapping was modified to specifically search against non-redundant NCBI database. From the LCMS/MS, protein identifications were validated with the following filters: protein score > 11, peptides score > 6 and scored peak intensity (SPI) > 60%. Only results with “Distinct Peptide” identification of 2 or greater than 2 are considered significant. Estimation of relative protein abundance through LCMS/MS technique was adapted from Aird *et al*.^36^ and Tan *et al*.^37^ based on the ratio of mean ion spectral intensity (MSI) of a protein to the total spectral intensity of all proteins in the chromatographic fraction.

### Immunological binding assay

Immunological binding activities between venom antigens and antivenoms were examined with an indirect enzyme-linked immunosorbent assay (ELISA) modified from Tan *et al*.^11^,^38^. In brief, immunoplate wells were each precoated overnight with 10 ng of different venoms (*N. sputatrix*, *B. fasciatus, C. rhodostoma*, *N. sumatrana, B. candidus*) at 4 °C. The plate was then flicked dry and rinsed four times with phosphate-buffered saline containing 0.5% Tween^®^20 (PBST). Antivenoms were prepared at a protein concentration of 40 mg/ml each, and 100 μl of appropriately diluted antivenom (1:6000) was added to each venom-coated well, followed by incubation for 1 h at room temperature. After washing the plate four times with PBST, 100 μl of appropriately diluted horseradish peroxidase-conjugated anti-horse-IgG (Jackson ImmunoResearch Inc., USA) in PBST (1:8000) was added to the well and incubated for another hour at room temperature. The excess components were removed by washing four times with PBST. A hundred microliters of freshly prepared substrate solution (0.5 mg/mL o-phenylenediamine and 0.003% hydrogen peroxide in 0.1 M citrate-phosphate buffer, pH 5.0) was then added to each well. The enzymatic reaction was allowed to take place in the dark for 30 min at room temperature. The reaction was subsequently terminated by adding 50 μl of 12.5% sulfuric acid, and the absorbance at 492 nm was read against the blank using an ELISA reader (SUNRISE-TECAN Type Touch Screen F039300, Switzerland). Immunological binding activity was expressed as percentage of relative absorbance between two comparing specific or paraspecific antivenoms and one non-specific antivenom (providing basal value for non-specific binding) toward the respective venoms. Values were means ± S.E.M. of triplicate experiments, and the difference between two comparing antivenoms was analyzed using unpaired t-test with *p* value < 0.05 for significance.

### Neutralization of venom procoagulant (thrombin-like enzyme) activity

Procoagulant activity of the venom was determined by adding 100 μl of *C. rhodostoma* venom of various concentrations in saline to 200 μl of bovine fibrinogen (2 g/l) according to a modified turbidimetric method^39^,^40^. The absorbance at 405 nm was monitored every 30 s over 30 min. This produced a plot of absorbance vs. time (min) with an initial lag time at which absorbance began to increase due to clot formation, followed by a hyperbolic curve and a plateau. The clotting time was determined as the time when the absorbance became 0.02 U greater than the mean of the first two absorbance measurements. The minimum clotting dose (MCD) was defined as the dose of venom that induces substrate coagulation in 5 min. In *in vitro* neutralization study, venom equivalent to 2 MCD was pre-incubated with various doses of the antivenoms (SABU or HPAV) at 37 °C for 30 min before adding to 200 μl bovine fibrinogen (2 g/l). The determination of clotting time in the neutralization assay was performed as above. The effective dose (ED) was defined as the dose of antivenom that prolonged the clotting time of bovine fibrinogen 3 times the control (2 MCD, without antivenom).

### Determination of venom lethality and neutralization by antivenom

The protocol of animal studies was approved by the Institutional Animal Care and Use Committee of the University of Malaya (Ethics clearance number: 2014–09–11/PHAR/R/TCH) and was based on the Council for International Organizations of Medical Sciences (CIOMS) guidelines on animal experimentation^41^. Median lethal doses (LD_50_) of venoms were determined by intravenous injection (via caudal veins) into ICR mice (*n* = 4 per dose). The survival ratio was recorded after 48 h. In antivenom neutralization assay, pre-incubation of venom and antivenom was conducted as described by Tan *et al*.^19^. Briefly, a challenge dose at 2.5 or 5 LD_50_ of the venom dissolved in normal saline was pre-incubated with various dilutions of antivenoms at 37 °C for 30 min. Subsequently, the mixture was injected intravenously into the mice (*n* = 4 per dose). The mice were allowed free access to food and water *ad libitum*, and the ratio of survival was recorded at 48 h post injection. Where 200 μl of reconstituted antivenom failed to give full protection to the mice, a lower lethal challenge dose (2.5 LD_50_) would be used. The neutralizing capacity of antivenom was expressed as ED_50_, defined as the amount of reconstituted antivenom that gives 50% survival in the venom-challenged animals. These parameters were determined according to the probit analysis method of Finney^42^ using BioStat 2009 analysis software (AnalystSoft Inc., Canada). The neutralization capacity was also expressed in term of ‘neutralization potency’ (P, defined as the amount of venom neutralized completely by a unit of antivenom) according to the recommendation by Morais *et al*.^28^,^43^. The neutralization potency is a more direct indicator of antivenom neutralizing capacity, and is theoretically unaffected by the number of LD_50_ in the challenge dose. For comparative purpose, the P values of different antivenoms were normalized by their respective protein amount and expressed as milligram of venom neutralized per gram of antivenom protein^27^.

## Additional Information

**How to cite this article**: Tan, C. H. *et al*. Assessing SABU (Serum Anti Bisa Ular), the sole Indonesian antivenom: A proteomic analysis and neutralization efficacy study. *Sci. Rep.* **6**, 37299; doi: 10.1038/srep37299 (2016).

**Publisher’s note:** Springer Nature remains neutral with regard to jurisdictional claims in published maps and institutional affiliations.

## Supplementary Material

Supplementary Information

## Figures and Tables

**Table 1:** Composition of Indonesian antivenom SABU (Serum Anti Bisa Ular) according to molecular mass as separated by size-exclusion fast protein liquid chromatography.

High Molecular mass proteins Molecular weight > 150 kDa		Major peak proteins Molecular weight ~100–120 kDa		Moderate/Low Molecular mass proteins Molecular weight < 60 kDa	
Protein	Percentage	Protein	Percentage	Protein	Percentage
Immunoglobulin chains	5.73	Immunoglobulin chains	84.04	Serum albumin	5.47
Serum albumin	0.71	Serum albumin	0.21	Immunoglobulin chains	1.96
Alpha-2-macroglobulin	0.28	Fibrinogen chain	0.21	Alpha-2-macroglobulin	0.17
Apolipoprotein-A	0.21	Apolipoprotein-A	0.03	Fibronectin	0.32
Alpha-1-antiproteinase	0.16			Serotransferrin	0.11
Haptoglobin	0.1			Plasminogen	0.10
Serotransferrin	0.07			Complement factor B	0.04
Fibrinogen chain	0.02			Inter-alpha-trypsin inhibitor	0.01
Inter-alpha-trypsin inhibitor	0.004			Kininogen	0.01
Antithrombin-III	0.003			Fibrinogen chain	0.004
Attractin	0.003			Carboxypeptidase	0.003
Total	7.27	Total	84.50	Total	8.2

**Table 2:**
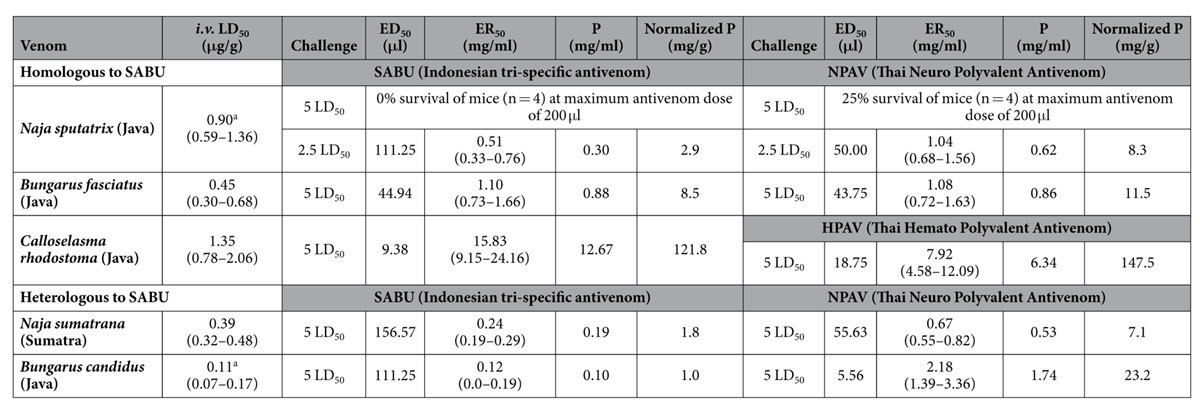
Neutralization of the lethal effect of five Indonesian snake venoms by different polyvalent antivenoms.

SABU: Serum Anti Bisa Ular; NPAV: Neuro Polyvalent Antivenom; *i.v*.: intravenous; LD_50_: median lethal dose; ED_50_: antivenom dose (μl) at which 50% of mice survived; ER_50_: antivenom dose (mg/ml) at which 50% of mice survived; P: potency expressed as the amount of venom neutralized by one ml antivenom; Normalized P was defined as the amount of venom (mg) neutralized per amount of antivenom protein (g).

^a^The two LD_50_ values were based on Leong *et al*.^17^ conducted in the same laboratory using the same venom stock.
