# Supplementary material for: Assessing SABU (Serum Anti Bisa Ular), the sole Indonesian antivenom: A proteomic analysis and neutralization efficacy study
Source: Sci Rep. 2016 Nov 21;6:37299. doi: 10.1038/srep37299 (PMC5116744; doi:10.1038/srep37299)
Supplement: Supplementary Information [file srep37299-s1.pdf]

Manuscript: Assessing SABU (Serum Anti Bisa Ular), the sole Indonesian antivenom: A proteomic analysis and neutralization efficacy study

Authors: Choo Hock Tan*, Jia Lee Liew, Kae Yi Tan, and Nget Hong Tan

Supplementary File Table S1. Mass spectrometric information of SABU (Serum Anti Bisa Ular) derived from LC-MS/MS.

Peaks 1 & 2

Database Accession	Protein Name	z	Score	Fwd-Rev Score	SPI (%)	Spectral Intensity	Sequence	RT (min)	Average Chi Squated	m/z Measured (Da)	MH+ Mass Shift (Da)	MH+ Error (ppm)	Protein % in a fraction
F6RI47	Uncharacterized protein	3	15.51	15.51	76.4	7.13E+04	(R)KYSNPSNCFGR(E)	4.6	0.99	443.8672	-0.0134	-10.1	4.74%
	(alpha-2-macroglobulin-like)	2	13.62	13.62	72.6	5.22E+04	(R)KYSNPSNCFGR(E)	4.6	1	665.2979	-0.0119	-9	
		2	18.8	18.8	83.4	3.72E+05	(K)YSNPSNCFGR(E)	4.95	0.99	601.2521	-0.0086	-7.1	
		3	15.33	15.33	95	3.79E+05	(R)EIHVSEPPTETVR(K)	5.3	0.99	498.5877	-0.0109	-7.3	
		2	18.12	18.12	99	1.82E+05	(R)EIHVSEPPTETVR(K)	5.3	0.99	747.3788	-0.0091	-6.1	
		2	16.65	16.65	74.3	6.31E+05	(K)FSQQLNNQGCLSQQVK(T)	5.55	1	939.9541	-0.0117	-6.3	
		2	21.1	12.35	87.5	5.05E+05	(K)IQEEGTEVELTGR(G)	5.6	0.98	730.8607	-0.0086	-5.9	
		2	15.31	4.56	81.3	4.53E+05	(R)IAQWQNLK(L)	5.83	0.99	500.779	-0.0067	-6.7	
		3	15.02	15.02	86.6	1.77E+05	(K)SFVHLEPMPR(E)	6.52	1	404.8749	-0.0092	-7.6	
		2	15.55	15.55	76.2	1.38E+05	(K)SFVHLEPMPR(E)	6.53	1	606.8098	-0.0071	-5.8	
		4	11.3	1.71	78.4	1.82E+04	(K)ESLVFVQTDKPIYKPGQTVK(F)	6.7	0.89	570.0652	-0.0099	-4.4	
		3	16.76	8.84	95.8	2.15E+05	(K)ESLVFVQTDKPIYKPGQTVK(F)	6.63	0.99	759.7523	-0.0066	-2.9	
		3	13.74	13.74	77.1	1.47E+05	(K)MVSGFVPLKPTVK(T)	6.83	1	468.273	-0.0082	-5.9	

		2	11.56	11.56	76.4	5.12E+04	(K)MVSGFVPLKPTVK(T)	6.83	1	701.9064	-0.0071	-5.1	
		2	15.16	5.84	87.9	6.17E+04	(R)EVLESLTEEAVK(E)	7.08	0.97	673.8528	-0.0066	-4.9	
		2	20.58	20.58	93.1	5.10E+04	(R)NVYINGIMYSPVSNTNEK(D)	7.37	0.86	1021.9881	-0.0162	-8	
		3	20.12	12.69	97.9	9.16E+05	(K)VDLSFSPAQSLPGSPAHLR(V)	7.47	0.99	660.346	-0.0111	-5.6	
		2	19.93	19.93	93.2	1.90E+05	(K)VDLSFSPAQSLPGSPAHLR(V)	7.47	0.98	990.0164	-0.009	-4.5	
		2	14.19	6.65	74.9	1.61E+05	(K)DIYSFLK(D)	7.9	1	443.2362	-0.0065	-7.4	
		2	21.9	21.9	87.5	2.02E+05	(K)AGALCLSSGAGLGLSPTASLR(A)	7.93	0.97	980.017	-0.0061	-3.1	
		3	16.47	8.57	84.6	1.03E+05	(K)AGALCLSSGAGLGLSPTASLR(A)	7.92	0.95	653.6791	-0.01	-5.1	
		2	19.67	13.53	100	3.44E+05	(R)VSVNLEESPAFLAVPGEK(E)	8.28	0.99	943.4953	-0.0072	-3.8	
		2	12.57	12.57	77.6	5.42E+05	(K)QLTFPLSSEPFQGSYK(V)	8.32	0.98	914.9558	-0.0073	-4	
		3	13.82	13.82	78.2	8.66E+04	(K)QLTFPLSSEPFQGSYK(V)	8.32	0.99	610.304	-0.0141	-7.7	
		2	15.31	15.31	88	3.37E+05	(R)LLVYTILPDGEVVGDSAK(Y)	8.5	0.99	945.013	-0.0079	-4.2	
		3	17.15	17.15	91.1	1.37E+05	(R)LLVYTILPDGEVVGDSAK(Y)	8.5	0.98	630.3431	-0.0119	-6.3	
		3	16.61	16.61	88.3	6.73E+03	(R)GHFSVSVLVESDIAPIAR(L)	8.45	0.61	633.0054	-0.0161	-8.5	
		3	15.38	15.38	94.1	5.15E+05	(R)AEHPFIVEEFVLPK(F)	8.63	0.99	552.2965	-0.009	-5.4	
		2	15.22	15.22	90.9	3.78E+05	(R)AEHPFIVEEFVLPK(F)	8.63	1	827.9426	-0.006	-3.6	
		2	19.34	19.34	81.3	6.59E+04	(R)NALFCLESAWK(S)	8.65	0.97	669.8265	-0.0054	-4	
		3	13.22	13.22	78	1.02E+06	(K)DLFHCVSFIVPR(A)	8.87	1	497.2555	-0.0101	-6.8	
		2	19.96	19.96	86.8	1.01E+06	(K)DLFHCVSFIVPR(A)	8.87	1	745.3795	-0.0103	-6.9	
		2	16.61	11.39	81.3	1.19E+06	(R)QGIPFFGQVLLVDGK(G)	10.02	0.99	809.4494	-0.0084	-5.2	
		3	12.85	12.85	75.3	1.54E+04	(R)QGIPFFGQVLLVDGK(G)	10.02	0.78	539.9667	-0.0144	-8.9	
		4	12.21	12.21	73	6.26E+03	(R)TYIFIDEAHITEALTWLSHK(Q)	10.07	0.61	597.8047	-0.0265	-11.1	
		3	15.96	7.5	85.4	5.24E+03	(R)TYIFIDEAHITEALTWLSHK(Q)	10.05	0.54	796.7397	-0.0189	-7.9	

		2	19.27	19.27	86.4	5.61E+03	(K)ELVFYYLMMAK(G)	10.17	0.99	704.3516	-0.0092	-6.5	
Q5XLE4	Serum albumin	3	16.31	16.31	81.9	7.05E+04	(K)YICEHQDSISGK(L)	4.4	0.97	479.548	-0.018	-12.5	6.96%
		2	19.77	19.77	96.5	1.32E+04	(K)YICEHQDSISGK(L)	4.42	0.86	718.8237	-0.0073	-5.1	
		2	19.48	19.48	94.1	4.09E+05	(K)EACFAEEGPK(L)	4.77	1	569.2441	-0.0072	-6.3	
		2	21.16	21.16	89.5	1.85E+06	(K)ADFTECCPADDK(A)	4.82	1	714.7697	-0.0085	-5.9	
		2	14.81	14.81	72.4	3.97E+05	(R)ATYGELADCCEK(Q)	5.12	0.99	708.7908	-0.0027	-1.9	
		3	18.73	18.73	97.8	7.22E+05	(K)ECCHGDLLECADDR(A)	5.35	0.97	583.8893	-0.0092	-5.2	
		2	24.66	24.66	90.7	6.85E+05	(K)ECCHGDLLECADDR(A)	5.35	0.97	875.3316	-0.0066	-3.8	
		3	22.41	13.63	94.1	1.88E+06	(K)LKPEPDAQCAAFQEDPD K(F)	5.43	0.99	686.9842	-0.0056	-2.7	
		2	14.12	8.96	76.3	1.72E+06	(K)LCTVATLR(A)	5.47	1	467.2607	-0.0045	-4.8	
		2	15.11	3.94	85.1	1.82E+06	(K)YLYEVAR(R)	5.6	1	457.2394	-0.0063	-6.9	
		3	12.01	5.97	86	9.50E+05	(K)SLHTLFGDK(L)	5.97	1	339.8474	-0.0087	-8.6	
		2	18.92	8.97	100	1.02E+06	(K)SLHTLFGDK(L)	5.97	1	509.2685	-0.0066	-6.5	
		3	22.31	22.31	96.1	6.98E+05	(K)LKPEPDAQCAAFQEDPD KFLGK(Y)	6.78	1	835.4062	-0.0086	-3.4	
		3	14.04	14.04	84.7	2.98E+05	(R)RHPDYSVSLLLR(I)	6.98	0.99	485.9374	-0.009	-6.2	
		2	14.41	10.22	78.4	1.19E+05	(R)HPDYSVSLLLR(I)	7.43	1	650.3526	-0.0076	-5.9	
		3	13.67	13.67	78.8	7.97E+04	(R)HPDYSVSLLLR(I)	7.43	0.97	433.9027	-0.012	-9.2	
		3	15.06	15.06	86.7	3.64E+05	(R)RHPYFYGPELLFHAEEY K(A)	7.73	0.98	766.0432	-0.0035	-1.5	
		4	10.95	2.85	70.8	1.70E+05	(R)HPYFYGPELLFHAEEYK(A)	8.42	0.99	535.7562	-0.0145	-6.8	
		3	16.31	16.31	81.3	2.01E+05	(K)CCAEADPPACYATVFDQ FTPLVEEPK(S)	9.45	0.99	1005.7732	-0.0159	-5.3	
		2	21.5	21.5	95	1.14E+04	(K)CCAEADPPACYATVFDQ FTPLVEEPK(S)	9.43	0.96	1508.156	-0.0162	-5.4	
		2	14.01	5.71	83.6	2.19E+06	(K)DVFLGTFLYEYSR(R)	9.8	0.98	805.3958	-0.0054	-3.3	
		3	14.86	7.78	88.2	4.56E+04	(K)DVFLGTFLYEYSR(R)	9.78	0.81	537.264	-0.0122	-7.6	

P35747	Serum albumin	3	16.31	16.31	81.9	7.05E+04	(K)YICEHQDSISGK(L)	4.4	0.97	479.548	-0.018	-12.5	7.27%
		2	19.77	19.77	96.5	1.32E+04	(K)YICEHQDSISGK(L)	4.42	0.86	718.8237	-0.0073	-5.1	
		2	19.48	19.48	94.1	4.09E+05	(K)EACFAEEGPK(L)	4.77	1	569.2441	-0.0072	-6.3	
		2	21.16	21.16	89.5	1.85E+06	(K)ADFTECCPADDK(L)	4.82	1	714.7697	-0.0085	-5.9	
		2	14.81	14.81	72.4	3.97E+05	(R)ATYGELADCCEK(Q)	5.12	0.99	708.7908	-0.0027	-1.9	
		3	18.73	18.73	97.8	7.22E+05	(K)ECCHGDLLECADDR(A)	5.35	0.97	583.8893	-0.0092	-5.2	
		2	24.66	24.66	90.7	6.85E+05	(K)ECCHGDLLECADDR(A)	5.35	0.97	875.3316	-0.0066	-3.8	
		3	22.41	13.63	94.1	1.88E+06	(K)LKPEPDAQCAAFQEDPD K(F)	5.43	0.99	686.9842	-0.0056	-2.7	
		2	14.12	8.96	76.3	1.72E+06	(K)LCTVATLR(A)	5.47	1	467.2607	-0.0045	-4.8	
		2	15.57	15.57	73.5	1.02E+05	(K)CSSFQNFGER(A)	5.57	0.95	616.2579	-0.0075	-6.1	
		2	15.11	3.94	85.1	1.82E+06	(K)YLYEVAR(R)	5.6	1	457.2394	-0.0063	-6.9	
		3	12.01	5.97	86	9.50E+05	(K)SLHTLFGDK(L)	5.97	1	339.8474	-0.0087	-8.6	
		2	18.92	8.97	100	1.02E+06	(K)SLHTLFGDK(L)	5.97	1	509.2685	-0.0066	-6.5	
		3	22.31	22.31	96.1	6.98E+05	(K)LKPEPDAQCAAFQEDPD KFLGK(Y)	6.78	1	835.4062	-0.0086	-3.4	
		4	12.15	12.15	74.8	8.13E+05	(R)RHPYFYGPELLFHAEEY K(A)	7.73	0.99	574.7834	-0.0068	-3	
		3	14.04	14.04	84.7	2.98E+05	(R)RHPDYSVSLLLR(I)	6.98	0.99	485.9374	-0.009	-6.2	
		2	14.41	10.22	78.4	1.19E+05	(R)HPDYSVSLLLR(I)	7.43	1	650.3526	-0.0076	-5.9	
		3	13.67	13.67	78.8	7.97E+04	(R)HPDYSVSLLLR(I)	7.43	0.97	433.9027	-0.012	-9.2	
		3	15.06	15.06	86.7	3.64E+05	(R)RHPYFYGPELLFHAEEY K(A)	7.73	0.98	766.0432	-0.0035	-1.5	
		4	10.95	2.85	70.8	1.70E+05	(R)HPYFYGPELLFHAEEYK(A)	8.42	0.99	535.7562	-0.0145	-6.8	
		2	14.01	5.71	83.6	2.19E+06	(K)DVFLGTFLYEYSR(R)	9.8	0.98	805.3958	-0.0054	-3.3	
		3	14.86	7.78	88.2	4.56E+04	(K)DVFLGTFLYEYSR(R)	9.78	0.81	537.264	-0.0122	-7.6	
P27425	Serotransferrin	3	18.18	18.18	88.1	1.28E+06	(K)AACVCQELHNQQASYG K(N)	5.03	0.99	655.293	-0.0105	-5.3	1.34%

		2	23.44	23.44	87.7	4.71E+05	(K)AACVCQELHNQQASYGK(N)	5.03	0.99	982.4366	-0.009	-4.6	
		3	16.63	16.63	82.4	1.22E+05	(R)LLEACTFHR(V)	5.37	0.98	382.8595	-0.0085	-7.4	
		2	26.5	26.5	100	1.06E+05	(K)CDEWSVNSGGNIECESAQSTEDCIAK(I)	6.57	1	1473.5889	-0.0117	-4	
		2	16.23	10.57	94.2	1.76E+05	(K)SSSDPDLTWNSLK(G)	6.92	1	725.3443	-0.0043	-3	
		2	22.65	14.18	94.3	1.62E+05	(R)TAVPNLCQLCVGK(G)	7.12	0.98	730.3707	-0.0055	-3.7	
		2	19.55	12.25	93.6	2.25E+05	(K)SIVPAPPLVACVK(R)	7.68	1	675.8917	-0.0052	-3.9	
		2	13.48	13.48	74.8	4.69E+05	(K)GEADAMSLDGGFIYIAGK(C)	8.75	0.96	907.9306	-0.009	-5	
		2	13.48	13.48	77.6	7.85E+03	(R)TAGWNIPMGLLYSEIK(H)	10.25	-0.34	896.9622	-0.0131	-7.3	
H9GZU9	Uncharacterized protein (Fragment)	2	15.91	15.91	95.4	1.62E+06	(K)ALPAPVER(T)	5.03	0.99	426.7481	-0.0049	-5.7	35.81%
	(Immunoglobulin heavy chain)	2	18.16	18.16	83.4	6.61E+06	(K)VSVTCLVK(D)	6	0.93	453.2579	-0.0039	-4.4	
		2	18.32	18.32	84.8	2.02E+05	(K)QGESFTCGVMHEAVENHYTQK(N)	6.23	0.99	1226.5331	-0.0067	-2.7	
		3	20.89	13.51	95.7	5.63E+06	(K)DFYPPEIDVEWQSNEHPEPEGK(Y)	7.82	0.99	881.3933	-0.0028	-1.1	
		3	17.2	9.81	95.5	2.49E+06	(R)VVSVLPIQHQDWLSGK(E)	7.73	1	602.6679	-0.0017	-0.9	
		2	19.85	16.28	95.1	7.51E+05	(R)VVSVLPIQHQDWLSGK(E)	7.73	0.99	903.4973	-0.0035	-2	
		2	23.63	16.4	97.2	9.34E+05	(K)DFYPPEIDVEWQSNEHPEPEGK(Y)	7.8	0.99	1321.587	-0.0014	-0.5	
		4	15.42	15.42	92.7	4.18E+05	(K)DFYPPEIDVEWQSNEHPEPEGK(Y)	7.82	1	661.295	-0.01	-3.8	
		2	17.78	17.78	91.8	9.69E+06	(K)APDVFPLTICGNTPDPK(V)	8	0.98	921.457	-0.0035	-1.9	
		3	14.8	14.8	77.4	2.70E+06	(K)APDVFPLTICGNTPDPK(V)	8	1	614.6388	-0.0084	-4.5	
		4	12.65	12.65	77.8	2.54E+06	(K)GDIHTFPLDLSNSAHHSLSSMMAVPR(S)	8.08	0.93	705.841	-0.0134	-4.8	
		3	25.17	25.17	100	7.16E+05	(K)GDIHTFPLDLSNSAHHSLSSMMAVPR(S)	8.08	0.94	940.786	-0.0121	-4.3	

		3	22.17	22.17	100	3.39E+07	(K)VPVGCLVSNYFPEPVTVSWNCDALK(G)	9.53	0.95	951.1298	-0.0045	-1.6	
		2	23.45	23.45	100	1.30E+07	(K)VPVGCLVSNYFPEPVTVSWNCDALK(G)	9.52	0.97	1426.1911	-0.0044	-1.6	
		4	10.88	10.88	74.7	6.28E+06	(K)VPVGCLVSNYFPEPVTVSWNCDALK(G)	9.53	0.98	713.5962	-0.0164	-5.7	
Q95M34	Immunoglobulin gamma 1 heavy chain constant region (Fragment)	2	14.79	14.79	74.2	1.87E+05	(R)IQHQDWLSGK(E)	5.33	1	606.3111	-0.0018	-1.5	3.47%
		2	14.23	14.23	87.5	6.24E+04	(K)VNNQALPQPIER(T)	5.63	0.94	689.8718	-0.0074	-5.4	
		2	18.16	18.16	83.4	6.61E+06	(K)VSVTCLVK(D)	6	0.93	453.2579	-0.0039	-4.4	
		2	21.15	21.15	84.5	2.10E+05	(K)YSTTQAQQDSDGSYFLYSK(L)	6.67	0.99	1094.9857	-0.0028	-1.3	
		2	22.2	22.2	100	2.93E+05	(R)TPEVTCVVVDVSQENPDVK(F)	7.23	1	1058.015	-0.0047	-2.2	
		3	16.75	16.75	88.3	2.92E+05	(R)TPEVTCVVVDVSQENPDVK(F)	7.23	1	705.6779	-0.0083	-3.9	
		2	17.04	17.04	78.4	1.47E+05	(K)FNWYMDGVEVR(T)	8	0.99	708.3216	-0.0053	-3.8	
		2	20.32	20.32	96	1.24E+04	(K)DFYPPEINIEWQSNGQPELETK(Y)	8.72	0.72	1317.6163	-0.0105	-4	
		2	13.7	13.7	77.9	6.75E+03	(K)CPAPELLGGPSVFIFPPNPK(D)	9.48	0.73	1069.0552	-0.0119	-5.6	
H9GZU8	Uncharacterized protein (Fragment)	2	20.82	20.82	92.6	3.20E+06	(K)SQTYICNVAHPASSTK(V)	5.03	1	882.4197	-0.006	-3.4	2.48%
	(Immunoglobulin heavy chain)	2	15.91	15.91	95.4	1.62E+06	(R)ALPAPVER(T)	5.03	0.99	426.7481	-0.0049	-5.7	
		2	22.2	22.2	100	2.93E+05	(R)TPEVTCVVVDVSQENPDVK(F)	7.23	1	1058.015	-0.0047	-2.2	
		3	16.75	16.75	88.3	2.92E+05	(R)TPEVTCVVVDVSQENPDVK(F)	7.23	1	705.6779	-0.0083	-3.9	
		4	14.19	2.89	75.2	1.44E+04	(K)LTVETDRWEQGESFTCVVMHEAIR(H)	8.28	0.97	724.09	-0.0226	-7.8	
		2	17.91	17.91	86	1.74E+05	(R)QSSGLYSLSSMVTVPASSLK(S)	8.73	0.97	1021.5236	-0.0075	-3.7	

F6Z2L5	Uncharacterized protein	2	12.52	12.52	71.2	1.11E+05	(K)AHPALEDLR(Q)	5.2	0.99	511.2713	-0.0072	-7	1.95%
	(Apolipoprotein A)	2	16.28	16.28	75.6	1.85E+05	(K)KWQEEVEVYR(Q)	5.72	0.99	683.3408	-0.0054	-4	
		3	16.53	16.53	78.7	7.33E+05	(K)LREQLGPVTQDFWDK(L)	7.43	0.99	611.3134	-0.0081	-4.4	
		2	20.17	20.17	95.4	1.23E+06	(K)VAPLSDEFR(E)	6.2	1	517.2677	-0.0032	-3.1	
		2	19.15	19.15	100	5.28E+05	(K)LREQLGPVTQDFWDK(L)	7.43	0.99	916.4672	-0.0066	-3.6	
		2	17.33	17.33	81.1	1.18E+06	(R)EQLGPVTQDFWDK(L)	7.63	0.99	781.8757	-0.0044	-2.8	
		2	16.39	9.22	85.9	5.87E+04	(R)VNLAPFSEELR(Q)	7.65	1	637.8378	-0.0056	-4.4	
		2	22.58	22.58	94.8	2.07E+05	(K)ASFLAAIDEASK(Q)	7.7	1	611.8175	-0.0037	-3	
		3	16.33	16.33	82.7	1.84E+05	(K)LREQLGPVTQDFWDKLEK(D)	7.92	0.97	734.7201	-0.0096	-4.4	
F7CSL8	Uncharacterized protein	2	15.47	15.47	80.6	6.45E+04	(K)QINDYVENGTQGK(I)	4.98	0.99	733.344	-0.011	-7.5	1.14%
	(alpha-antiproteinease)	2	18.5	18.5	87	1.93E+05	(K)GDTHTQILEGLR(F)	6.08	0.97	670.3491	-0.0055	-4.1	
		2	19.24	11.09	89.5	4.36E+05	(K)LSISGCYDLK(R)	6.53	0.99	578.2863	-0.0061	-5.3	
		2	18.6	18.6	94.9	3.92E+05	(K)GTEAAGATIVEAIR(T)	6.97	0.99	679.8645	-0.0057	-4.2	
		2	17.5	17.5	93.5	6.91E+05	(R)ILPELGITK(V)	7.13	1	492.3078	-0.0052	-5.3	
		3	15.67	15.67	83.4	1.39E+05	(K)NLYHSEAFSINFGDIEEAK(K)	8.2	0.94	728.6763	-0.01	-4.6	
		2	15.76	15.76	84.8	9.33E+03	(K)NLYHSEAFSINFGDIEEAK(K)	8.2	0.65	1092.5122	-0.0073	-3.3	
		2	18.01	18.01	91.6	6.23E+05	(K)GILATFLENR(H)	8.52	1	567.3157	-0.0072	-6.4	
		4	12.48	5.32	74.1	1.67E+04	(R)TLLHTNVEFNRPFVLIIYDR(N)	9.07	0.98	615.8345	-0.0236	-9.6	
		3	12.75	12.75	93.2	5.42E+03	(R)TLLHTNVEFNRPFVLIIYDR(N)	9.07	0.59	820.7775	-0.0218	-8.9	
B5BV12	Alpha-1-antitrypsin	2	15.47	15.47	80.6	6.45E+04	(K)QINDYVENGTQGK(I)	4.98	0.99	733.344	-0.011	-7.5	0.14%
		2	16.1	16.1	89.6	1.12E+05	(R)QADLSGITEEAPLTVSK(A)	7.08	0.99	879.9557	-0.0079	-4.5	

		3	15.67	15.67	83.4	1.39E+05	(K)NLYHSEAFSINFGDIEEAK(K)	8.2	0.94	728.6763	-0.01	-4.6	
		2	15.76	15.76	84.8	9.33E+03	(K)NLYHSEAFSINFGDIEEAK(K)	8.2	0.65	1092.5122	-0.0073	-3.3	
H9GZT5	Uncharacterized protein (Fragment)	2	20.82	20.82	92.6	3.20E+06	(K)SQTYICNVAHPASSTK(V)	5.03	1	882.4197	-0.006	-3.4	7.09%
	(Immunoglobulin heavy chain)	2	15.91	15.91	95.4	1.62E+06	(K)ALPAPVER(T)	5.03	0.99	426.7481	-0.0049	-5.7	
		2	18.16	18.16	83.4	6.61E+06	(K)VSVTCLVK(D)	6	0.93	453.2579	-0.0039	-4.4	
		2	14.57	14.57	80.7	1.06E+06	(R)VVSILAIQHK(D)	6.3	1	554.3453	-0.0051	-4.6	
		3	18.8	18.8	92.5	5.26E+05	(K)ECGGCPTCPECLSVGPSVFIFPPKPK(D)	8.62	0.92	974.1176	-0.0118	-4	
		2	22.6	22.6	91.8	2.85E+06	(K)DFYPTDIDIEWK(S)	8.62	1	771.3594	-0.0043	-2.8	
		3	16.68	10.06	97.4	1.14E+05	(K)DFYPTDIDIEWK(S)	8.62	0.96	514.5731	-0.0111	-7.2	
H9GZS9	Uncharacterized protein (Fragment)	3	15.93	15.93	90.4	7.06E+05	(K)TYICNVAHPASSTK(V)	4.78	0.99	516.9166	-0.0123	-7.9	4.21%
	(Immunoglobulin heavy chain)	2	22.4	22.4	92.6	3.43E+05	(K)TYICNVAHPASSTK(V)	4.78	0.99	774.8734	-0.008	-5.2	
		2	18.16	18.16	83.4	6.61E+06	(K)VSVTCLVK(D)	6	0.93	453.2579	-0.0039	-4.4	
		3	12.91	12.91	77.9	4.27E+04	(R)VPQVYVLAPHPDELSK(N)	7.12	0.89	597.9886	-0.0127	-7.1	
		3	16.28	8.01	86.8	3.81E+04	(K)DFYPTDITVEWQSNEHPEPEGK(Y)	7.77	0.79	873.3931	-0.0034	-1.3	
		3	10.8	10.8	71.6	7.64E+03	(K)CPAPELLGGPSVFIFPPKPK(D)	8.98	0.87	717.7232	-0.012	-5.6	
		3	16.61	13.33	85.3	1.37E+06	(R)TFPSVLQSSGLYSLSSMVTVPASSLESK(T)	10.1	1	968.1539	-0.0283	-9.7	
		2	19.55	11.23	94.3	3.84E+05	(R)TFPSVLQSSGLYSLSSMVTVPASSLESK(T)	10.1	0.97	1451.731	-0.0207	-7.1	
P38029	Alpha-1-antiproteinase 2	2	16.43	16.43	79.9	3.89E+05	(K)LQHLEDTLTK(G)	5.22	0.99	599.325	-0.0047	-3.9	1.82%
		2	10.8	1.75	71.8	7.94E+04	(K)FLEDVK(K)	5.3	0.96	375.7016	-0.0073	-9.7	
		2	16.44	4.5	93.2	4.63E+05	(K)AVLTIDEK(G)	5.43	1	444.7529	-0.0052	-5.8	

		2	12.53	1.51	89.7	7.20E+05	(K)IVDLVK(D)	5.48	1	343.7232	-0.0056	-8.1	
		2	16.78	8.81	93.2	1.37E+06	(K)DTVLALVNYIFFK(G)	11.9	0.99	771.9245	-0.0149	-9.7	
		3	17.78	9.37	89.8	1.09E+06	(K)DLDKDTVLALVNYIFFK(G)	11.97	0.91	672.0285	-0.0186	-9.2	
H9GZN9	Uncharacterized protein (Fragment)	2	19.59	19.59	91.9	3.91E+05	(R)LVCQATGFSPK(E)	5.43	0.97	604.3085	-0.0042	-3.5	0.77%
	(Immunoglobulin heavy chain)	2	10.73	2.03	76.5	8.52E+04	(K)EISVSWLR(D)	7.65	1	495.2713	-0.0061	-6.2	
		2	18.2	18.2	92.4	6.45E+05	(K)GFSPPDVFVQWLQK(G)	10.05	0.97	824.4271	-0.006	-3.7	
		3	18.46	18.46	92.1	1.32E+04	(K)GFSPPDVFVQWLQK(G)	10.03	0.78	549.9507	-0.0154	-9.4	
		3	17.66	8.39	88.3	5.31E+05	(K)TPDLFPLVSCGPSLDESLVAVGCLAR(D)	10.47	0.98	925.1254	-0.0283	-10.2	
		2	13.55	2.58	80	7.81E+04	(K)IFAIPPSFAGIFLTK(S)	10.7	0.92	811.4635	-0.0155	-9.6	
A0A0B4J1C4	Uncharacterized protein	2	14.45	14.45	76.1	1.58E+05	(R)VPLTYGGQTK(I)	4.93	0.99	532.2886	-0.0083	-7.8	0.37%
	(Immunoglobulin J)	2	14.22	6.41	84.6	1.96E+05	(R)IIIPVNSR(E)	6	1	456.2844	-0.0058	-6.3	
		3	18.41	18.41	84.9	1.74E+05	(K)FVYHLSDLCK(K)	6.55	0.98	427.8784	-0.009	-7	
		2	24.01	24.01	97.6	3.06E+05	(R)IIPSPENPNEDILER(H)	6.83	0.97	868.4442	-0.005	-2.9	
F6V5H1	Uncharacterized protein (Fragment)	3	15.35	9.05	86.7	2.57E+05	(K)TSSFHSSLTEQDSK(D)	4.63	0.98	518.5717	-0.0073	-4.7	1.97%
	(Immunoglobulin light chain)	2	21.49	21.49	97	1.83E+05	(K)TSSFHSSLTEQDSK(D)	4.63	0.96	777.3513	-0.0125	-8	
		2	23.01	23.01	93.7	3.74E+05	(K)ADYEAHNVYACEVSHK(T)	4.88	0.99	946.9098	-0.0109	-5.7	
		2	11.44	11.44	74.5	2.40E+06	(K)TLSSPLVK(S)	5.55	1	422.7573	-0.0065	-7.7	
		2	13.71	13.71	80.6	1.22E+06	(K)DNTYSLSSTLTLPK(A)	7.57	0.99	770.3967	-0.004	-2.6	
A0A0A1E3W4	Immmunoglobulin lambda light chain variable	2	15.76	15.76	80.3	1.01E+05	(K)GNGAAISQGVQTTKPSK(Q)	4.4	0.91	822.4301	-0.0182	-11.1	3.59%

	region (Fragment)												
		3	14.3	9.42	74.5	8.87E+05	(K)SYSSVSCQVTHQGK(T)	4.5	1	523.2401	-0.0112	-7.1	
		2	21.56	21.56	88.1	4.06E+05	(K)SYSSVSCQVTHQGK(T)	4.5	0.99	784.3569	-0.0104	-6.6	
		3	13.86	13.86	72.1	6.66E+06	(K)ATVVCLISDFSPSDLTVS WK(G)	10.18	1	742.3726	-0.0126	-5.7	
		2	21.42	16.47	100	5.09E+04	(K)ATVVCLISDFSPSDLTVS WK(G)	10.08	0.98	1113.0569	-0.0093	-4.2	
A0A0A1E4 70	Immmunoglobu lin lambda light chain variable region (Fragment)	2	11.51	5.73	71.8	5.78E+04	(K)SYSSVSCQVK(H)	4.47	0.99	572.7643	-0.009	-7.8	0.15%
		2	16.64	16.64	85.8	1.13E+05	(K)VGWFQQIPGTAPK(T)	7.7	0.98	714.8826	-0.0055	-3.8	
		2	18	18	95.1	1.77E+05	(K)ATVVCLISDFSPSGLEVI WK(V)	10.73	1	1111.075	-0.0146	-6.6	
A0A0A1E6 I1	Immmunoglobu lin lambda light chain variable region (Fragment)	2	18.39	18.39	88.4	1.54E+05	(R)LTIAGGPTSTPSVSLFPPS SEELSANK(A)	8.38	0.9	1344.1868	-0.0111	-4.1	3.04%
		3	13.86	13.86	72.1	6.66E+06	(K)ATVVCLISDFSPSDLTVS WK(V)	10.18	1	742.3726	-0.0126	-5.7	
		2	21.42	16.47	100	5.09E+04	(K)ATVVCLISDFSPSDLTVS WK(V)	10.08	0.98	1113.0569	-0.0093	-4.2	
A0A0A1E3 Y7	Immmunoglobu lin lambda light chain variable region (Fragment)	3	16.81	16.81	79.6	9.61E+05	(K)VNGAAISQGVQTTKPSK(Q)	4.63	0.97	562.6419	-0.0069	-4.1	3.40%
		3	13.86	13.86	72.1	6.66E+06	(K)ATVVCLISDFSPSDLTVS WK(V)	10.18	1	742.3726	-0.0126	-5.7	
		2	21.42	16.47	100	5.09E+04	(K)ATVVCLISDFSPSDLTVS WK(V)	10.08	0.98	1113.0569	-0.0093	-4.2	

A0A0A1E6A8	Immmunoglobulin lambda light chain variable region (Fragment)	2	14	14	77.6	5.24E+04	(K)YAASSYLPLTPTQWK(S)	8.2	0.89	863.4462	0.0005	0.3	3.00%
		3	13.86	13.86	72.1	6.66E+06	(K)ATVVCLISDFSPSDLTVSWK(V)	10.18	1	742.3726	-0.0126	-5.7	
		2	21.42	16.47	100	5.09E+04	(K)ATVVCLISDFSPSDLTVSWK(V)	10.08	0.98	1113.0569	-0.0093	-4.2	
H9GZV1	Uncharacterized protein (Fragment)	3	13.49	13.49	81.4	5.82E+04	(R)LSGKPTHVNVSVVMAEADGTCY(-)	7.02	0.99	779.0354	-0.014	-6	0.63%
	(Immunoglobulin heavy chain)	2	12.07	12.07	74.7	9.05E+05	(R)EPGQSVPTFAVTSLLR(V)	9.02	0.97	851.4558	-0.0127	-7.5	
		3	15.24	15.24	86.5	1.32E+05	(R)EPGQSVPTFAVTSLLR(V)	9.02	0.92	567.9727	-0.0135	-7.9	
		2	20.06	20.06	95.7	3.21E+05	(K)GFFPLGFPEPVK(V)	9.47	0.96	667.8556	-0.0104	-7.8	
Q7SIH1	Alpha-2-macroglobulin	2	13.82	4.66	86.6	8.64E+04	(R)DLKPAIVK(V)	5.07	1	442.2803	-0.0078	-8.8	0.80%
		2	18.6	10.33	90.5	7.82E+05	(R)VTASPQSLCALR(A)	6.12	1	651.8448	-0.0011	-0.9	
		3	12.77	6.67	87	5.41E+05	(K)DTIIKPLLVEPEGLEK(E)	7.92	0.98	598.6772	-0.0088	-4.9	
		2	12.11	4.93	75.4	3.91E+05	(K)DTIIKPLLVEPEGLEK(E)	7.92	0.99	897.5142	-0.0048	-2.7	
P0DM92	Apolipoprotein A-I (Fragment)	2	17.09	9.54	84.8	2.54E+05	(R)EYVAQFEASALGK(Q)	7.07	1	706.8537	-0.0055	-3.9	0.44%
		4	10.85	4.6	80.2	1.63E+05	(K)LREQIGPVTQEFWDNLEK(E)	7.92	0.97	551.2895	0.0172	7.8	
		3	10.7	0	79.3	4.06E+05	(R)EQIGPVTQEFWDNLEK(E)	8.27	0.97	644.9918	0.0271	14	
		2	15.9	6.78	74.7	1.60E+05	(R)EQIGPVTQEFWDNLEK(E)	8.27	0.97	966.9854	0.0297	15.4	
P0DMA9	Apolipoprotein A-I	3	10.7	0	79.3	4.06E+05	(R)EQLGPVTQEFWDNLEK(E)	8.27	0.97	644.9918	0.0271	14	1.09%
		2	15.9	6.78	74.7	1.60E+05	(R)EQLGPVTQEFWDNLEK(E)	8.27	0.97	966.9854	0.0297	15.4	
		2	11.67	11.67	70.7	1.91E+06	(R)QGLLPVLESLK(V)	9.15	1	598.8628	-0.0066	-5.5	

F6XWM5	Uncharacterized protein (Fragment)	2	17.43	17.43	91.9	7.79E+05	(K)YVVTLPVADQDTCVK(H)	6.63	1	804.898	-0.0051	-3.1	1.99%
	(Haptoglobulin)	2	19.58	19.58	94.6	3.70E+06	(K)VPSILDWVQK(T)	8.85	1	592.8339	-0.0069	-5.8	
F6R3F4	Uncharacterized protein (Fragment)	2	18.57	18.57	94.5	1.67E+05	(R)LGVYELLLK(V)	8.78	1	524.3219	-0.0083	-8	0.08%
	(Inter-alpha-trypsin inhibitor heavy chain)	2	16.66	16.66	83.8	1.93E+04	(K)VTFELVYEELLK(R)	9.92	0.99	741.903	-0.0103	-6.9	
F6RUZ6	Fibrinogen alpha chain	2	18.46	18.46	85.1	1.64E+05	(K)GLIDEVNQDFTNR(I)	7.42	0.99	760.8676	-0.006	-4	0.10%
		2	16.12	16.12	75.8	7.12E+04	(K)ETDWPFCSDEDWNYK(C)	8.25	0.97	996.3863	-0.0099	-5	
P32262	Antithrombin-III	2	17.55	11.45	85.9	5.00E+04	(R)DIPVNPMCIYR(S)	7.68	0.97	689.3329	-0.0068	-5	0.05%
		2	15.69	9.13	77.8	5.73E+04	(R)VAEGTQVLELPFK(G)	7.97	0.92	715.8933	-0.0096	-6.7	
F6W2Y1	Uncharacterized protein	2	14.86	14.86	79.9	1.33E+05	(R)DNCCILDER(F)	5.55	0.98	597.7437	-0.0077	-6.4	0.12%
	(Fibrinogen gamma chain)	2	17.89	17.89	86.1	7.21E+04	(K)DLQDFEDILHR(A)	8.08	0.96	700.8425	-0.0027	-2	
		3	14.38	9.51	76	5.72E+04	(K)DLQDFEDILHR(A)	8.08	0.95	467.5619	-0.0093	-6.7	
													100.00%

Peak 3

Database Accession	Protein Name	z	Score	Fwd-Rev Score	SPI (%)	Spectral Intensity	Sequence	RT (min)	Average Chi Squated	m/z Measured (Da)	MH+ Mass Shift (Da)	MH+ Error (ppm)	Mean Spectral (protein % in a fraction)
P35747	Serum albumin	3	16.17	16.17	84.6	1.00E+06	(K)YICEHQDSISGK(L)	4.43	0.99	479.5497	-0.0129	-9	2.52%
		2	24.76	24.76	96.9	2.11E+05	(K)YICEHQDSISGK(L)	4.43	0.99	718.8218	-0.0111	-7.7	
		2	19.62	19.62	88.1	6.01E+05	(K)ADFTECCPADDK(L)	4.92	0.99	714.77	-0.0079	-5.5	
		2	14.08	14.08	80.3	5.58E+04	(K)EACFAEEGPK(L)	4.85	1	569.2445	-0.0064	-5.6	
		2	16.36	16.36	79.2	1.04E+05	(R)ATYGELADCCEK(Q)	5.27	0.99	708.787	-0.0103	-7.2	
		3	18.07	18.07	95.4	4.96E+05	(K)ECCHGDLLECADDR(A)	5.48	0.98	583.8879	-0.0134	-7.6	
		2	23.36	23.36	90.4	2.77E+05	(K)ECCHGDLLECADDR(A)	5.48	0.98	875.33	-0.0098	-5.6	
		3	20.78	12.32	85.2	3.19E+05	(K)LKPEPDAQCAAFQEDPDK(F)	5.57	0.97	686.9826	-0.0104	-5.1	
		2	12.09	4.04	75.5	6.85E+05	(K)LCTVATLR(A)	5.6	1	467.2584	-0.0091	-9.7	
		2	12.65	4.91	81.1	6.84E+05	(K)YLYEVAR(R)	5.78	1	457.2384	-0.0083	-9.1	
		3	12.2	5.94	89.1	4.45E+05	(K)SLHTLFGDK(L)	6.25	1	339.8468	-0.0105	-10.3	
		2	16.59	6.51	87.1	3.43E+05	(K)SLHTLFGDK(L)	6.25	0.99	509.2677	-0.0082	-8.1	
		3	19.36	19.36	89.4	3.10E+05	(K)LKPEPDAQCAAFQEDPDKFLGK(Y)	6.88	1	835.4048	-0.0128	-5.1	
		3	13.04	13.04	79.7	8.97E+04	(R)RHPDYSVSLLLR(I)	7.12	0.96	485.9372	-0.0096	-6.6	

		4	11.92	11.92	76.5	4.78E+05	(R)RHPYFYGPELLFHAEEYK(A)	7.87	0.99	574.782	-0.0124	-5.4	
		2	16.99	8.9	86.8	2.25E+05	(K)DVFLGTFLYEYSR(R)	9.82	0.87	805.393	-0.011	-6.8	
F6RI47	Uncharacterized protein	2	15.3	15.3	73.6	7.17E+04	(K)FSQQLNNQGCLSQQVK(T)	5.67	0.91	939.9528	-0.0143	-7.6	0.76%
	(Alpha-2-macroglobulin-like protein)	2	10.55	2.68	71.1	1.57E+05	(R)IAQWQNLK(L)	6.03	0.99	500.7787	-0.0073	-7.3	
		3	14.46	6.22	89.8	6.29E+04	(K)ESLVFVQTDKPIYKPGQTVK(F)	6.73	0.98	759.7502	-0.0129	-5.6	
		3	17.98	9.96	93.5	2.74E+05	(K)VDLSFSPAQSLPGSPAHLR(V)	7.57	0.98	660.3452	-0.0135	-6.8	
		2	17.88	17.88	80.1	2.76E+04	(K)VDLSFSPAQSLPGSPAHLR(V)	7.57	0.95	990.0146	-0.0126	-6.4	
		2	12.66	5.1	74	4.80E+04	(K)DIYSFLK(D)	7.97	1	443.2357	-0.0075	-8.5	
		2	13.47	13.47	72.8	7.52E+03	(K)AGALCLSSGAGLGLSPTASLR(A)	7.97	0.67	980.0127	-0.0147	-7.5	
		2	18.55	14.02	97.7	9.39E+04	(R)VSVNLEESPAFLAVPGEK(E)	8.33	0.9	943.4937	-0.0104	-5.5	
		2	14.52	14.52	82.8	1.62E+05	(K)QLTFPLSSEPFQGSYK(V)	8.38	0.96	914.9555	-0.0079	-4.3	
		3	11.63	11.63	73.2	6.96E+04	(R)ELPCGQTQTVQVHYILNGQVLQELK(E)	8.47	0.93	965.8322	-0.0213	-7.3	
		3	15.93	15.93	82.4	5.13E+04	(R)LLVYTILPDGEVVGDSAK(Y)	8.53	0.87	630.3411	-0.0179	-9.5	
		3	15.4	15.4	95	1.11E+05	(R)AEHPFIVEEFVLPK(F)	8.7	0.98	552.2961	-0.0102	-6.1	
		2	14.95	10.22	89.6	6.99E+04	(R)AEHPFIVEEFVLPK(F)	8.7	0.98	827.9423	-0.0066	-4	
		3	11.92	11.92	83.3	1.77E+05	(K)DLFHCVSFIVPR(A)	8.88	1	497.2557	-0.0095	-6.4	
		2	20.19	20.19	93.7	1.54E+05	(K)DLFHCVSFIVPR(A)	8.88	1	745.3811	-0.0071	-4.8	
		2	17.34	13.79	90.2	3.88E+05	(R)QGIPFFGQVLLVDGK(G)	10.03	0.95	809.4498	-0.0076	-4.7	
H9GZT5	Uncharacterized protein (Fragment)	2	20.27	20.27	91.9	2.74E+06	(K)SQTYICNVAHPASSTK(V)	5.15	0.99	882.4179	-0.0096	-5.4	2.85%
		2	16.38	16.38	94.9	7.94E+05	(K)ALPAPVER(T)	5.15	1	426.7465	-0.0081	-9.4	
		2	18.2	10.51	83.8	1.88E+06	(K)VSVTCLVK(D)	6.2	0.99	453.2569	-0.0059	-6.6	

		3	18.02	18.02	81.1	1.01E+05	(R)EPQVYVLAPHRDELSK(N)	6.2	0.99	627.6623	-0.0141	-7.5	
		2	13.48	13.48	78.4	3.16E+05	(R)VVSILAIQHK(D)	6.58	1	554.343	-0.0097	-8.8	
		2	21.81	21.81	87.7	6.42E+05	(K)YSTTPAQLDSDGSYFLYSK(L)	7.65	1	1071.9933	-0.0073	-3.4	
		3	17.14	17.14	84.9	1.51E+05	(K)ECGGCPTCPECLSVGPSVFIFPPKPK(D)	8.62	0.94	974.115	-0.0196	-6.7	
		2	22.69	22.69	93.8	5.51E+05	(K)DFYPTDIDIEWK(S)	8.65	1	771.3591	-0.0049	-3.2	
		3	12.82	9.28	81.1	1.42E+04	(K)DFYPTDIDIEWK(S)	7.67	0.11	514.5733	-0.0105	-6.8	
H9GZU8	Uncharacterized protein (Fragment)	2	20.27	20.27	91.9	2.74E+06	(K)SQTYICNVAHPASSTK(V)	5.15	0.99	882.4179	-0.0096	-5.4	1.48%
	(Immunoglobulin heavy chain)	2	16.38	16.38	94.9	7.94E+05	(R)ALPAPVER(T)	5.15	1	426.7465	-0.0081	-9.4	
		2	18.41	18.41	88.4	2.87E+04	(R)TPEVTCVVVDVSQENPDVK(F)	7.32	0.96	1058.0114	-0.0119	-5.6	
		3	11.78	7.19	75.7	4.39E+04	(R)TPEVTCVVVDVSQENPDVK(F)	7.32	0.92	705.6754	-0.0158	-7.5	
		2	17.5	17.5	85.6	1.15E+05	(R)QSSGLYSLSSMVTVPASSLK(S)	8.75	1	1021.5249	-0.0049	-2.4	
H9GZQ9	Uncharacterized protein (Fragment)	2	18.2	10.51	83.8	1.88E+06	(K)VSVTCLVK(D)	6.2	0.99	453.2569	-0.0059	-6.6	0.78%
	(Immunoglobulin heavy chain)	2	19.85	19.85	86.8	7.86E+03	(K)YSTTQAQQDSDGSYFLYSK(L)	6.73	0.64	1094.9818	-0.0106	-4.8	
		2	18.41	18.41	88.4	2.87E+04	(R)TPEVTCVVVDVSQENPDVK(F)	7.32	0.96	1058.0114	-0.0119	-5.6	
		3	11.78	7.19	75.7	4.39E+04	(R)TPEVTCVVVDVSQENPDVK(F)	7.32	0.92	705.6754	-0.0158	-7.5	
H9GZU9	Uncharacterized protein (Fragment)	2	16.38	16.38	94.9	7.94E+05	(K)ALPAPVER(T)	5.15	1	426.7465	-0.0081	-9.4	34.53%
	(Immunoglobulin heavy chain)	2	18.2	10.51	83.8	1.88E+06	(K)VSVTCLVK(D)	6.2	0.99	453.2569	-0.0059	-6.6	
		3	17.22	10.03	95.8	1.06E+06	(R)VVSVLPIQHQDWLSGK(E)	7.87	0.99	602.6658	-0.008	-4.4	

		3	20.28	12.97	94	7.53E+05	(K)DFYPPEIDVEWQSNEHPEPEGK(Y)	7.88	0.99	881.3917	-0.0076	-2.9	
		2	20.29	13.7	94.3	9.97E+04	(K)DFYPPEIDVEWQSNEHPEPEGK(Y)	7.87	0.94	1321.5829	-0.0096	-3.6	
		2	19.27	19.27	91.7	1.05E+07	(K)APDVFPLTICGNTPDPK(V)	8.05	0.95	921.4582	-0.0011	-0.6	
		3	15.06	15.06	77.8	3.64E+06	(K)APDVFPLTICGNTPDPK(V)	8.05	1	614.639	-0.0078	-4.2	
		4	11.03	11.03	76.3	2.01E+06	(K)GDIHTFPLDLSNSAHHSLSSMMAVPR(S)	8.13	0.9	705.8406	-0.015	-5.3	
		3	24.17	24.17	100	4.49E+05	(K)GDIHTFPLDLSNSAHHSLSSMMAVPR(S)	8.13	0.95	940.7865	-0.0106	-3.8	
		3	18.41	18.41	96.9	4.21E+07	(K)VPVGCLVSNYFPEPVTVSWNCDALK(G)	9.55	0.95	951.1305	-0.0024	-0.8	
		2	23.45	23.45	100	2.02E+07	(K)VPVGCLVSNYFPEPVTVSWNCDALK(G)	9.55	0.96	1426.1931	-0.0004	-0.1	
		4	15.53	10.08	87.4	5.28E+06	(K)VPVGCLVSNYFPEPVTVSWNCDALK(G)	9.55	0.98	713.5972	-0.0124	-4.3	
F6Z2L5	Uncharacterized protein	2	16.54	16.54	82.4	5.02E+04	(K)KWQEEVEVYR(Q)	5.92	1	683.3421	-0.0028	-2.1	0.79%
	(Apoliproprotein A1)	2	20.49	20.49	96.8	7.40E+05	(K)VAPLSDEFR(E)	6.42	0.99	517.2649	-0.0088	-8.5	
		3	16.6	16.6	83.8	1.58E+05	(K)LREQLGPVTQDFWDK(L)	7.55	0.99	611.3124	-0.0111	-6	
		2	17.3	17.3	96.1	6.50E+04	(K)LREQLGPVTQDFWDK(L)	7.55	0.98	916.4641	-0.0128	-7	
		2	17.25	17.25	81.9	3.26E+05	(R)EQLGPVTQDFWDK(L)	7.73	0.99	781.8743	-0.0072	-4.6	
		2	20.69	20.69	90.5	1.38E+05	(K)ASFLAAIDEASK(Q)	7.83	1	611.8162	-0.0063	-5.1	
		2	22	22	93.3	5.18E+05	(K)LLDNWDSLGTTLGK(L)	8.28	0.99	766.8979	-0.007	-4.6	
H9GZS9	Uncharacterized protein (Fragment)	3	15.94	15.94	90.6	4.33E+06	(K)TYICNVAHPASSTK(V)	4.88	0.99	516.9169	-0.0114	-7.3	4.61%
	(Immunoglobulin heavy chain)	2	25.51	25.51	94.4	1.82E+06	(K)TYICNVAHPASSTK(V)	4.88	0.99	774.8728	-0.0092	-5.9	
		2	11.51	11.51	73.1	3.46E+04	(K)VNNQALPAPVER(T)	5.48	0.98	654.3511	-0.0117	-8.9	
		2	18.2	10.51	83.8	1.88E+06	(K)VSVTCLVK(D)	6.2	0.99	453.2569	-0.0059	-6.6	
		2	13.02	13.02	75.1	2.31E+05	(K)DSDGSYFLYSK(L)	6.75	0.99	641.2849	-0.0009	-0.7	

		3	14.48	9.33	84	7.85E+03	(R)TFPSVLQSSGLYSLSSmV TVPASSLESK(T)	9.2	0.71	973.4915	15.9845	-3.6	
		3	13.16	13.16	70.8	1.08E+05	(R)TFPSVLQSSGLYSLSSMV TVPASSLESK(T)	10.02	0.98	968.1612	-0.0064	-2.2	
		2	23.67	14.54	98.7	2.62E+06	(R)TFPSVLQSSGLYSLSSMV TVPASSLESK(T)	10.1	0.99	1451.7391	-0.0045	-1.5	
		4	14.04	14.04	81.9	5.78E+05	(R)TFPSVLQSSGLYSLSSMV TVPASSLESK(T)	10.1	1	726.3716	-0.0108	-3.7	
O75882	Attractin	2	17.46	17.46	80.1	8.69E+04	(K)GDECQLCEVENR(Y)	5.28	0.93	754.804	-0.0097	-6.4	0.09%
		2	18.52	12.78	84.8	2.19E+04	(R)SEAACLAAGPGIR(C)	6.13	1	636.8187	-0.0064	-5	
		2	16.8	16.8	86.2	1.05E+04	(R)LTGSSGFVTDGPGNYK(Y)	6.05	0.81	800.3828	-0.0066	-4.1	
		2	19.61	12.08	90.3	6.42E+03	(K)LTLTPWVGLR(K)	8.83	0.75	578.3438	-0.0081	-7	
		3	14	14	76.2	1.12E+05	(R)NQECIALPENICGIGWHL VGNSCLK(I)	8.98	0.86	961.464	0.0028	1	
P27425	Serotransferrin	3	15.66	15.66	84.1	3.44E+05	(K)AACVCQELHNQQASYG K(N)	5.15	0.98	655.2916	-0.0147	-7.5	0.23%
		2	22.46	22.46	87	7.14E+04	(K)AACVCQELHNQQASYG K(N)	5.15	0.98	982.434	-0.0142	-7.2	
		3	20.05	20.05	84.5	4.95E+04	(K)CDEWSVNSGGNIECESA QSTEDCIAK(I)	6.65	0.85	982.7276	-0.014	-4.8	
		2	24.04	24.04	84.9	2.40E+04	(K)CDEWSVNSGGNIECESA QSTEDCIAK(I)	6.65	0.98	1473.5883	-0.0129	-4.4	
		2	13.39	6.09	78.2	2.47E+04	(K)SIVPAPPLVACVK(R)	7.8	0.9	675.8895	-0.0096	-7.1	
		2	13.48	13.48	83.8	5.73E+04	(K)GEADAMSLDGGFIYIAG K(C)	8.63	0.92	907.9341	-0.002	-1.1	
A0A0A1E3 V9	Immmunoglobu lin lambda light chain variable region (Fragment)	3	12.58	8.49	80.5	8.91E+05	(K)GNGAAISQGVQTTKPSK(Q)	4.43	0.96	548.6227	-0.0176	-10.7	10.21%
		2	15.68	15.68	86	1.57E+05	(K)GNGAAISQGVQTTKPSK(Q)	4.43	0.89	822.4317	-0.015	-9.1	
		3	14.32	9.26	75	1.30E+06	(K)SYSSVSCQVTHQGK(T)	4.55	0.99	523.2401	-0.0112	-7.1	
		2	20.46	20.46	85.5	4.20E+05	(K)SYSSVSCQVTHQGK(T)	4.55	0.99	784.357	-0.0102	-6.5	

		2	16.76	16.76	84.9	7.44E+03	(K)LTQPSSVSVALGQTATITCK(G)	7.2	0.67	1031.5394	-0.0134	-6.5	
		2	20.19	15.43	97.3	8.21E+06	(K)ATVVCLISDFSPSDLTVSWK(G)	10.17	1	1113.0571	-0.0089	-4	
		3	16.72	16.72	86.8	1.47E+07	(K)ATVVCLISDFSPSDLTVSWK(G)	10.18	1	742.3732	-0.0108	-4.9	
A0A0A1E6P5	Immmunoglobulin lambda light chain variable region (Fragment)	3	17.22	17.22	78.4	1.51E+06	(K)VNGAAISQGVQTTKPSK(Q)	4.72	0.99	562.6407	-0.0105	-6.2	9.73%
		2	13.82	8.73	80.2	7.86E+03	(R)LSVLGGPTSAPSVSLFPPSSEELSANK(A)	8.75	0.73	1336.1911	-0.0076	-2.8	
		3	15.54	7.77	86	2.36E+04	(R)LSVLGGPTSAPSVSLFPPSSEELSANK(A)	8.8	0.88	891.13	-0.0071	-2.6	
		2	20.19	15.43	97.3	8.21E+06	(K)ATVVCLISDFSPSDLTVSWK(V)	10.17	1	1113.0571	-0.0089	-4	
		3	16.72	16.72	86.8	1.47E+07	(K)ATVVCLISDFSPSDLTVSWK(V)	10.18	1	742.3732	-0.0108	-4.9	
A0A0A1E927	Immmunoglobulin lambda light chain variable region (Fragment)	2	16.76	16.76	84.9	7.44E+03	(K)LTQPSSVSVALGQTATITCK(G)	7.2	0.67	1031.5394	-0.0134	-6.5	9.14%
		2	15.42	15.42	87.6	8.13E+04	(K)YAASSYLPLTPTQWK(S)	8.28	0.95	863.4455	-0.0009	-0.5	
		2	20.19	15.43	97.3	8.21E+06	(K)ATVVCLISDFSPSDLTVSWK(V)	10.17	1	1113.0571	-0.0089	-4	
		3	16.72	16.72	86.8	1.47E+07	(K)ATVVCLISDFSPSDLTVSWK(V)	10.18	1	742.3732	-0.0108	-4.9	
A0A0A1E417	Immmunoglobulin lambda light chain variable region (Fragment)	3	13.1	9.3	79.7	8.01E+05	(K)DSERPSGIPDR(F)	4.47	0.97	410.198	-0.0122	-9.9	3.65%
		2	14.27	6.93	88.6	1.75E+05	(K)DSERPSGIPDR(F)	4.47	0.98	614.7903	-0.0183	-14.9	
		2	15.49	9.42	79.8	1.06E+06	(K)SYSSVSCQVK(H)	4.5	0.99	572.7648	-0.008	-7	

		2	22.17	22.17	100	7.15E+06	(K)ATVVCLISDFSPSGLEVIWK(V)	10.82	1	1111.0763	-0.012	-5.4	
A0A0A1E6K2	Immmunoglobulin lambda light chain variable region (Fragment)	2	15.49	9.42	79.8	1.06E+06	(K)SYSSVSCQVK(H)	4.5	0.99	572.7648	-0.008	-7	3.27%
		3	10.45	10.45	81.2	3.48E+04	(K)TLIYDDTKR(A)	4.92	0.87	375.5328	-0.0108	-9.6	
		2	22.17	22.17	100	7.15E+06	(K)ATVVCLISDFSPSGLEVIWK(V)	10.82	1	1111.0763	-0.012	-5.4	
A0A0A1E6I1	Immmunoglobulin lambda light chain variable region (Fragment)	3	13.55	8.68	81.9	7.92E+05	(R)LTIAGGPTSTPSVSLFPPSSEELSANK(A)	8.43	0.96	896.4595	-0.0135	-5	9.58%
		2	14.41	14.41	87.9	3.86E+05	(R)LTIAGGPTSTPSVSLFPPSSEELSANK(A)	8.43	0.94	1344.1857	-0.0133	-4.9	
		2	20.19	15.43	97.3	8.21E+06	(K)ATVVCLISDFSPSDLTVSWK(V)	10.17	1	1113.0571	-0.0089	-4	
		3	16.72	16.72	86.8	1.47E+07	(K)ATVVCLISDFSPSDLTVSWK(V)	10.18	1	742.3732	-0.0108	-4.9	
F6V5H1	Uncharacterized protein (Fragment)	3	17.6	11.56	87.5	1.06E+06	(K)TSSFHSSLTEQDSK(D)	4.72	0.99	518.5707	-0.0103	-6.6	2.76%
	(Immunoglobulin light chain)	2	22.24	22.24	96.1	4.84E+05	(K)TSSFHSSLTEQDSK(D)	4.72	0.97	777.3527	-0.0097	-6.2	
		3	17.52	17.52	74.9	1.15E+06	(K)ADYEAHNVYACEVSHK(T)	5	1	631.609	-0.0107	-5.7	
		2	24	24	93.7	2.15E+05	(K)ADYEAHNVYACEVSHK(T)	4.98	0.99	946.9101	-0.0103	-5.4	
		2	11.01	11.01	75.3	2.36E+06	(K)TLSSPLVK(S)	5.7	1	422.7573	-0.0065	-7.7	
		2	14.01	14.01	82.3	1.69E+06	(K)DNTYSLSSTLTLPK(A)	7.65	0.99	770.3953	-0.0068	-4.4	
F6W2Y1	Uncharacterized protein	2	15.44	15.44	81.1	3.95E+05	(R)DNCCILDER(F)	5.65	0.98	597.7428	-0.0095	-7.9	0.35%

	(Fibrinogen gamma chain)	3	15.09	11.19	79.1	1.71E+05	(K)DLQDFEDILHR(A)	8.15	0.98	467.5619	-0.0093	-6.7	
		2	20.31	15.13	91.6	1.48E+05	(K)DLQDFEDILHR(A)	8.15	0.98	700.8369	-0.0139	-10	
		3	18.17	18.17	80.8	1.08E+05	(R)FGSYCPTTCGIADFLSNY QTSVDK(D)	9.17	0.97	911.069	-0.009	-3.3	
		2	26.97	26.97	92	5.31E+04	(R)FGSYCPTTCGIADFLSNY QTSVDK(D)	9.18	0.89	1366.1011	-0.0065	-2.4	
P0DM92	Apolipoprotein A-I (Fragment)	2	16.67	16.67	70.9	1.37E+06	(K)VQPYLDDFQK(K)	6.65	1	626.8106	-0.0069	-5.5	0.66%
		2	17.15	10.69	85.9	8.97E+04	(R)EYVAQFEASALGK(Q)	7.15	0.99	706.8524	-0.0081	-5.7	
		3	11.9	3.77	74.2	1.22E+05	(K)LREQIGPVTQEFWDNLE K(E)	8	0.99	734.7193	0.0244	11.1	
		2	16.09	7.3	75.7	7.70E+04	(R)EQIGPVTQEFWDNLEK(E)	8.33	0.93	966.9836	0.0261	13.5	
P0DMA9	Apolipoprotein A-I	2	16.09	7.3	75.7	7.70E+04	(R)EQLGPVTQEFWDNLEK(E)	8.33	0.93	966.9836	0.0261	13.5	0.36%
		2	12.84	12.84	74	8.32E+05	(R)QGLLPVLESLK(V)	9.2	1	598.8636	-0.005	-4.2	
P38029	Alpha-1-antiproteinase 2	2	10.75	10.75	72.4	6.39E+04	(K)QINDYVEK(G)	4.82	0.87	504.7488	-0.0093	-9.2	0.43%
		2	14.72	3.73	87.7	2.21E+05	(K)AVLTIDEK(G)	5.6	1	444.7515	-0.008	-8.9	
		2	16.51	9.97	87.5	3.50E+05	(K)DTVLALVNYIFFK(G)	11.88	0.92	771.9249	-0.0141	-9.2	
		3	17.95	10.13	92.2	4.32E+05	(K)DLDKDTVLALVNYIFFK(G)	11.98	0.88	672.0297	-0.015	-7.5	
F6XWM5	Uncharacterized protein (Fragm	3	19.3	19.3	97.8	8.56E+04	(K)LPECEAVCGKPK(N)	4.67	0.97	463.2238	-0.014	-10.1	0.29%
	(Haptoglobulin)	2	12.54	12.54	75.6	1.48E+04	(K)LPECEAVCGKPK(N)	4.67	0.99	694.332	-0.0141	-10.2	
		2	14.69	14.69	86.7	1.66E+05	(K)YVTLPVADQDTCVK(H)	6.73	0.99	804.8965	-0.0081	-5	
		2	14.89	14.89	81.9	4.55E+05	(K)VPSILDWVQK(T)	8.88	1	592.8344	-0.0059	-4.9	
P04937	Fibronectin	3	17.26	7.91	90.2	5.90E+04	(K)LGVRPSQGGEAPR(E)	4.42	0.99	441.9036	-0.0165	-12.5	0.14%
		3	12.82	6.74	81.2	4.94E+04	(R)VDVLPVNLPGEHGQR(L)	7.23	0.92	543.9574	-0.0131	-8	

		2	15.92	5.54	86.2	2.23E+05	(R)VTWAPPPSIELTNLLVR(Y)	10.07	0.94	953.5396	-0.0077	-4.1	
		3	18.32	18.32	85.8	8.76E+03	(R)VTWAPPPSIELTNLLVR(Y)	10.07	0.91	636.0256	-0.0174	-9.1	
F7CYR1	Uncharacterized protein	3	10.15	5.97	73.9	3.44E+04	(K)ATEDEGSEQKIPEATNR(R)	4.53	0.86	625.6245	-0.0137	-7.3	0.03%
	(Anti-thrombin III)	2	18.26	18.26	91.7	3.77E+04	(R)DIPVNPMCIYR(S)	7.78	1	689.332	-0.0086	-6.3	
		3	19.4	10.18	100	5.17E+03	(R)ITDVIPHGAINELTVLVLVNTIYFK(G)	11.73	0.65	928.1882	-0.0253	-9.1	
F7CYP1	Uncharacterized protein	2	11.71	11.71	70.8	2.13E+05	(K)LSISGCYDLK(R)	6.63	0.98	578.2861	-0.0065	-5.6	0.25%
	(Alpha-1-antiproteinase-like 2-like isoform)	2	16.65	9.2	96.1	1.21E+05	(K)GTEAAGATIVEAIR(T)	7.07	0.98	679.8623	-0.0101	-7.4	
		2	15.44	15.44	90.8	3.01E+05	(R)ILPELGITK(V)	7.25	1	492.3067	-0.0074	-7.6	
H9GZN9	Uncharacterized protein (Fragment)	2	16.61	16.61	91.8	7.67E+04	(R)LVCQATGFSPK(E)	5.57	0.99	604.3054	-0.0104	-8.7	0.05%
	(Immunoglobulin heavy chain)	2	13.4	4.66	78.7	2.75E+04	(K)EISVSWLR(D)	7.77	0.92	495.2693	-0.0101	-10.2	
		2	10.47	0.01	77.6	2.29E+04	(K)IFAIPPSFAGIFLTK(S)	10.68	0.8	811.4648	-0.0129	-8	
A0A0B4J1C4	Uncharacterized protein	3	16.73	16.73	82.7	6.23E+04	(K)FVYHLSDLCK(K)	6.67	0.98	427.8771	-0.0129	-10	0.03%
	(Immunoglobulin J chain)	2	18.87	18.87	94	1.51E+04	(R)IIPSPENPNEDILER(H)	6.93	0.98	868.4408	-0.0118	-6.8	
Q7SIH1	Alpha-2-macroglobulin	2	19.4	12.33	92.9	1.95E+05	(R)VTASPQSLCALR(A)	6.27	1	651.8417	-0.0073	-5.6	0.16%
		3	14.9	6.16	86.3	1.42E+05	(K)DTIIKPLLVEPEGLEK(E)	7.98	0.95	598.6765	-0.0109	-6.1	
		2	11.39	5.28	79.9	7.21E+04	(K)DTIIKPLLVEPEGLEK(E)	7.98	0.9	897.5124	-0.0084	-4.7	

H9GZV1	Uncharacterized protein (Fragment)	3	15.15	15.15	87.3	2.76E+05	(R)EPGQSVPTFAVTSLLR(V)	9.05	0.94	567.9708	-0.0192	-11.3	0.27%
	(Immunoglobulin heavy chain)	2	18.58	18.58	93.4	4.08E+05	(K)GFFPLGFPEPVK(V)	9.48	0.99	667.8575	-0.0066	-5	
													100.00%

Peak 4

Database Accession	Protein Name	z	Score	Fwd-Rev Score	SPI (%)	Spectral Intensity	Sequence	RT (min)	Average Chi Squated	m/z Measured (Da)	MH+ Mass Shift (Da)	MH+ Error (ppm)	Mean Spectral (protein % in a fraction)
P35747	Serum albumin	2	18.02	18.02	86.9	2.02E+05	(K)ADFTECCPADDK(L)	4.77	0.98	714.7726	-0.0027	-1.9	0.25%
		2	23.3	23.3	94.2	9.07E+04	(K)ECCHGDLLECADDR(A)	5.28	0.96	875.3311	-0.0076	-4.3	
		2	12.85	2.06	75.1	2.64E+05	(K)LCTVATLR(A)	5.3	0.99	467.2595	-0.0069	-7.4	
		3	15.12	15.12	79.2	7.81E+04	(K)ECCHGDLLECADDR(A)	5.28	0.86	583.8876	-0.0143	-8.2	
		3	12.75	6.45	90.8	1.04E+05	(K)SLHTLFGDK(L)	5.85	1	339.848	-0.0069	-6.8	
		3	18.18	18.18	90	2.84E+05	(K)LKPEPDAQCAAFQEDPDKFLGK(Y)	6.67	1	835.4072	-0.0056	-2.2	
		4	10.7	10.7	70.9	9.17E+04	(R)RHPYFYGPELLFHAEEYK(A)	7.55	0.99	574.7824	-0.0108	-4.7	
		3	13.86	13.86	82.7	4.52E+04	(R)RHPYFYGPELLFHAEEYK(A)	7.53	0.9	766.043	-0.0041	-1.8	
		3	11.99	2.8	70.8	5.30E+04	(R)HPYFYGPELLFHAEEYK(A)	8.27	0.94	714.0092	-0.0044	-2.1	

H9GZU9	Uncharacterized protein (Fragment)	2	16.71	16.71	92.5	1.81E+05	(K)ALPAPVER(T)	4.92	1	426.7464	-0.0083	-9.7	40.22%
	(Immunoglobulin heavy chain)	2	13.01	13.01	74.5	2.31E+05	(K)VSVTCLVK(D)	5.85	1	453.2571	-0.0055	-6.1	
		3	19.28	12.59	97.8	1.60E+05	(R)VVSVLPIQHQDWLSGK(E)	7.57	0.99	602.6668	-0.005	-2.8	
		2	19.26	19.26	91.6	1.69E+07	(K)APDVFPLTICGNTPDPK(V)	7.85	0.9	921.4606	0.0037	2	
		3	15.11	15.11	78.7	3.00E+07	(K)APDVFPLTICGNTPDPK(V)	7.77	1	614.6407	-0.0027	-1.4	
		3	18.87	18.87	93.9	1.26E+06	(K)GDIHTFPLDLSNSAHHSLSSMMAVPR(S)	7.95	0.92	940.7894	-0.0019	-0.7	
		3	21.42	21.42	100	1.31E+08	(K)VPVGCLVSNYFPEPVTVSWNCDALK(G)	9.4	0.94	951.1311	-0.0006	-0.2	
		4	14.21	6.26	82.2	1.58E+07	(K)VPVGCLVSNYFPEPVTVSWNCDALK(G)	9.4	0.97	713.596	-0.0172	-6	
H9GZT5	Uncharacterized protein (Fragment)	2	19.54	19.54	92.1	1.49E+07	(K)SQTYICNVAHPASSTK(V)	4.97	1	882.4201	-0.0052	-2.9	3.20%
	(Immunoglobulin heavy chain)	2	16.71	16.71	92.5	1.81E+05	(K)ALPAPVER(T)	4.92	1	426.7464	-0.0083	-9.7	
		2	13.01	13.01	74.5	2.31E+05	(K)VSVTCLVK(D)	5.85	1	453.2571	-0.0055	-6.1	
		3	15.91	15.91	79.9	8.62E+04	(R)EPQVYVLAPHRDELSK(N)	5.87	0.99	627.6639	-0.0093	-5	
		2	14.5	14.5	81.5	7.31E+04	(K)YSTTPAQLDSDGSYFLYSK(L)	7.43	0.97	1071.9963	-0.0013	-0.6	
H9GZU8	Uncharacterized protein (Fragment)	2	19.54	19.54	92.1	1.49E+07	(K)SQTYICNVAHPASSTK(V)	4.97	1	882.4201	-0.0052	-2.9	3.16%
	(Immunoglobulin heavy chain)	2	16.71	16.71	92.5	1.81E+05	(R)ALPAPVER(T)	4.92	1	426.7464	-0.0083	-9.7	
		2	19.54	13.78	97.7	6.63E+04	(R)IVIKEPCCCPK(C)	5.02	0.93	702.3409	-0.0099	-7	
		2	16.54	16.54	86.9	1.43E+05	(R)QSSGLYSLSSMVTVPASSLK(S)	8.6	0.99	1021.5253	-0.0041	-2	

F6V5H1	Uncharacterized protein (Fragment)	3	17.32	17.32	86.6	1.38E+05	(K)TSSFHSSLTEQDSK(D)	4.55	0.98	518.5707	-0.0103	-6.6	2.85%
	(Immunoglobulin light chain)	2	20.13	20.13	95	7.37E+04	(K)TSSFHSSLTEQDSK(D)	4.55	0.97	777.3542	-0.0067	-4.3	
		3	16.99	16.99	76.8	3.41E+06	(K)ADYEAHNVYACEVSHK(T)	4.8	0.99	631.6113	-0.0038	-2	
		2	23.3	23.3	94	1.14E+06	(K)ADYEAHNVYACEVSHK(T)	4.8	1	946.9128	-0.0049	-2.6	
		2	14.61	8.26	91.4	4.54E+06	(K)TLSSPLVK(S)	5.38	0.96	422.7591	-0.0029	-3.5	
		2	14.03	14.03	81.9	4.52E+06	(K)DNTYSLSSTLTLPK(A)	7.45	1	770.398	-0.0014	-0.9	
A0A0A1E470	Immmunoglobulin lambda light chain variable region (Fragment)	2	12.56	6.75	76.9	1.42E+05	(K)SYSSVSCQVK(H)	4.5	0.99	572.7643	-0.009	-7.8	5.78%
		2	20.83	20.83	92.5	5.50E+06	(K)VNDAVTTDGVQTTR(S)	4.6	0.86	738.8633	-0.0096	-6.5	
		2	15.79	15.79	84	2.43E+05	(K)VGWFQQIPGTAPK(T)	7.53	0.99	714.884	-0.0027	-1.9	
		2	17.22	12.15	93.5	2.21E+07	(K)ATVVCLISDFSPSGLEVIWK(V)	10.77	1	1111.0759	-0.0128	-5.8	
A0A0A1E3V9	Immmunoglobulin lambda light chain variable region (Fragment)	2	19.61	19.61	89.1	2.41E+05	(K)SYSSVSCQVTHQGK(T)	4.48	0.98	784.3562	-0.0118	-7.5	14.67%
		2	20.34	20.34	87.2	1.99E+05	(K)LTQPSSVSVALGQTATITCK(G)	7.13	0.88	1031.5421	-0.008	-3.9	
		3	15.08	15.08	76.2	1.46E+05	(K)LTQPSSVSVALGQTATITCK(G)	7.12	0.88	688.0303	-0.0085	-4.1	
		2	21.72	15.97	100	2.59E+07	(K)ATVVCLISDFSPSDLTVSWK(G)	10.13	1	1113.0601	-0.0029	-1.3	
		3	16.54	16.54	83.1	3.82E+07	(K)ATVVCLISDFSPSDLTVSWK(G)	10.12	1	742.3729	-0.0117	-5.3	
		3	15.24	15.24	80	6.44E+06	(K)ATVVCLISDFSPSDLTVSWK(G)	11.8	0.85	742.3737	-0.0093	-4.2	

A0A0A1E6I1	Immmunoglobulin lambda light chain variable region (Fragment)	3	16.21	16.21	70.7	6.39E+05	(K)YAASSYLSLTPSQWK(S)	7.6	0.99	567.9527	-0.0047	-2.8	14.79%
		2	18.3	15.2	93.3	5.93E+05	(R)LTIAGGPTSTPSVSLFPPSSEELSANK(A)	8.27	0.98	1344.1935	0.0023	0.9	
		2	21.72	15.97	100	2.59E+07	(K)ATVVCLISDFSPSDLTVSWK(V)	10.13	1	1113.0601	-0.0029	-1.3	
		3	16.54	16.54	83.1	3.82E+07	(K)ATVVCLISDFSPSDLTVSWK(V)	10.12	1	742.3729	-0.0117	-5.3	
		3	15.24	15.24	80	6.44E+06	(K)ATVVCLISDFSPSDLTVSWK(V)	11.8	0.85	742.3737	-0.0093	-4.2	
A0A0A1E6P5	Immmunoglobulin lambda light chain variable region (Fragment)	2	18.73	18.73	86.1	3.32E+05	(K)VNGAAISQGVQTTKPSK(Q)	4.57	0.98	843.4592	-0.007	-4.1	14.60%
		2	13.29	13.29	77.9	9.65E+03	(R)LSVLGGPTSAPSVSLFPPSSEELSANK(A)	8.62	0.77	1336.1928	-0.0042	-1.6	
		2	21.72	15.97	100	2.59E+07	(K)ATVVCLISDFSPSDLTVSWK(V)	10.13	1	1113.0601	-0.0029	-1.3	
		3	16.54	16.54	83.1	3.82E+07	(K)ATVVCLISDFSPSDLTVSWK(V)	10.12	1	742.3729	-0.0117	-5.3	
		3	15.24	15.24	80	6.44E+06	(K)ATVVCLISDFSPSDLTVSWK(V)	11.8	0.85	742.3737	-0.0093	-4.2	
F6W2Y1	Uncharacterized protein	2	13.65	13.65	76.5	5.61E+04	(R)DNCCILDER(F)	5.42	0.98	597.7446	-0.0059	-4.9	0.25%
	(Fibrinogen chain)	3	13.93	8.46	73.7	3.68E+05	(K)DLQDFEDILHR(A)	7.97	0.99	467.5626	-0.0072	-5.2	
		2	18.68	18.68	88.8	4.41E+05	(K)DLQDFEDILHR(A)	7.97	0.98	700.8413	-0.0051	-3.7	
		2	28.57	28.57	96.1	1.47E+05	(R)FGSYCPTTCGIADFLSNYQTSVDK(D)	9.03	0.97	1366.1032	-0.0023	-0.8	
		3	15.66	15.66	71.9	2.08E+05	(R)FGSYCPTTCGIADFLSNYQTSVDK(D)	9.03	0.99	911.0707	-0.0039	-1.4	

H9GZS9	Uncharacterized protein (Fragment)	3	15.12	15.12	88.9	4.50E+05	(K)TYICNVAHPASSTK(V)	4.68	0.99	516.9182	-0.0075	-4.8	0.20%
	(Immunoglobulin light chain)	2	17.99	17.99	89	2.81E+05	(K)TYICNVAHPASSTK(V)	4.68	0.99	774.8737	-0.0074	-4.8	
		2	13.01	13.01	74.5	2.31E+05	(K)VSVTCLVK(D)	5.85	1	453.2571	-0.0055	-6.1	
F6Z2L5	Uncharacterized protein	2	13.42	13.42	76.9	6.83E+04	(K)VAPLSDEFR(E)	6.1	0.99	517.2707	0.0028	2.7	0.04%
	(Apolipoprotein A-1)	2	16.62	9.2	80	1.21E+05	(K)LLDNWDSLGTTLGK(L)	8.12	0.98	766.9	-0.0028	-1.8	
													100.00%

Peak 5

Database Accession	Protein Name	z	Score	Fwd-Rev Score	SPI (%)	Spectral Intensity	Sequence	RT (min)	Average Chi Squated	m/z (Da)	MH+ Mass Shift (Da)	MH+ Error (ppm)	Mean Spectral (protein % in a fraction)
P35747	Serum albumin	3	12.21	12.21	70.2	1.53E+05	(K)YICEHQDSISGK(L)	4.4	0.97	479.5479	-0.0183	-12.7	33.19%
		2	17.83	17.83	77.6	4.42E+03	(K)YICEHQDSISGK(L)	4.4	0.6	718.822	-0.0107	-7.5	
		2	21.12	21.12	89.2	1.83E+06	(K)ADFTECCPADDK(L)	4.65	0.92	714.78	0.0121	8.5	
		3	14.78	14.78	71.6	4.00E+05	(K)ADFTECCPADDK(L)	4.57	0.99	476.8503	-0.0043	-3	
		2	18.64	18.64	82.1	2.50E+06	(R)ATYGELADCCEK(Q)	4.75	0.95	708.7914	-0.0015	-1	
		3	15.55	4.68	91.4	2.65E+05	(K)QEPERNECFLTHKDDHPNLPK(L)	4.75	1	868.7468	-0.0001	0	

		2	23.81	23.81	89.1	1.37E+07	(K)ECCHGDLLECADDR(A)	5.02	0.95	875.3341	-0.0016	-0.9	
		3	18.72	18.72	97.6	1.30E+07	(K)ECCHGDLLECADDR(A)	5.02	0.95	583.8912	-0.0035	-2	
		2	13.75	13.75	75.3	6.41E+05	(K)LCTVATLR(A)	4.93	1	467.2613	-0.0033	-3.5	
		2	17.04	10.18	81.4	2.58E+05	(K)CSSFQNFGER(A)	4.98	1	616.2599	-0.0035	-2.9	
		3	22.65	13.72	100	4.16E+06	(K)LKPEPDAQCAAFQEDPD K(F)	5.2	0.92	686.9867	0.0019	0.9	
		2	24.79	17.11	93.6	7.29E+06	(K)LKPEPDAQCAAFQEDPD K(F)	5.27	0.99	1029.9761	0.0012	0.6	
		4	12.48	12.48	82	3.44E+05	(K)ECCHGDLLECADDRADL AK(Y)	5.6	0.98	562.7397	-0.0058	-2.6	
		3	20.62	20.62	100	2.60E+05	(K)ECCHGDLLECADDRADL AK(Y)	5.62	0.98	749.9848	-0.0029	-1.3	
		3	12.12	6.43	79.7	1.30E+06	(K)SLHTLFGDK(L)	5.67	1	339.8482	-0.0063	-6.2	
		2	19.02	9.11	92.9	1.32E+06	(K)SLHTLFGDK(L)	5.68	1	509.2697	-0.0042	-4.2	
		2	21.5	21.5	92.4	4.28E+05	(K)ECCHGDLLECADDRADL AK(Y)	5.78	0.99	1124.4721	-0.0058	-2.6	
		3	13.54	13.54	85	2.16E+05	(R)RHPDYSVSLLLR(I)	6.38	0.99	485.9382	-0.0066	-4.6	
		2	20.06	20.06	92	1.44E+06	(K)LKPEPDAQCAAFQEDPD KFLGK(Y)	6.48	1	1252.61	0.0001	0	
		2	13.31	6.02	80.9	8.64E+04	(R)RHPDYSVSLLLR(I)	6.38	0.98	728.4056	-0.0027	-1.9	
		5	11.55	3.48	78.4	5.65E+05	(K)LKPEPDAQCAAFQEDPD KFLGK(Y)	6.52	0.99	501.6464	-0.0097	-3.9	
		3	13.57	13.57	78.6	4.63E+04	(R)HPDYSVSLLLR(I)	7.23	1	433.9035	-0.0096	-7.4	
		3	12.7	7.63	82.9	4.23E+04	(R)RPCFSALELDEGYVPK(E)	7.2	0.9	627.6464	0.0035	1.9	
		2	15.58	15.58	88.8	1.50E+06	(R)RHPYFYGPELLFHAEEY K(A)	7.57	0.99	1148.562	-0.0018	-0.8	
		3	16.4	16.4	83.2	1.12E+07	(R)HPYFYGPELLFHAEEYK(A)	8.23	0.99	714.0094	-0.0038	-1.8	
		2	14.44	4.88	84.4	2.22E+05	(K)TVLGNFSAFVAK(C)	8.13	1	627.3468	-0.0025	-2	
		2	18.3	11.08	75.4	9.28E+05	(R)HPYFYGPELLFHAEEYK(A)	8.22	0.99	1070.5112	-0.0023	-1.1	
		3	14.66	8.32	87.4	8.13E+05	(K)EDDLPSDLPALAADFAE DKEICK(H)	9.02	1	854.7339	-0.0044	-1.7	
		2	17.21	13.11	94	1.09E+04	(K)EDDLPSDLPALAADFAE DKEICK(H)	9.02	0.79	1281.5988	-0.0013	-0.5	

		2	18.87	18.87	96.7	1.34E+05	(K)EDDLPSDLPALAADFAEDK(E)	9.32	0.96	1016.4719	-0.0028	-1.4	
		3	13.66	13.66	79.8	5.85E+03	(K)EDDLPSDLPALAADFAEDK(E)	9.32	0.59	677.9821	-0.0076	-3.7	
		2	16.91	9.8	84.6	3.76E+06	(K)DVFLGTFLYEYSR(R)	9.85	0.99	805.397	-0.003	-1.8	
		3	15.07	6.64	86.7	4.44E+05	(K)DVFLGTFLYEYSR(R)	9.83	0.88	537.2648	-0.0098	-6.1	
		3	18.17	7.09	92.6	1.38E+06	(K)GLVLVAFSQYLQQCPFEDHVK(L)	9.87	1	826.7528	-0.0047	-1.9	
		4	14.34	2.68	82.3	3.30E+05	(K)GLVLVAFSQYLQQCPFEDHVK(L)	9.87	0.99	620.3161	-0.006	-2.4	
		2	13.32	6.23	76.1	1.16E+05	(K)GLVLVAFSQYLQQCPFEDHVK(L)	9.87	0.92	1239.6219	-0.0121	-4.9	
Q5XLE4	Serum albumin	3	12.21	12.21	70.2	1.53E+05	(K)YICEHQDSISGK(L)	4.4	0.97	479.5479	-0.0183	-12.7	33.56%
		2	17.83	17.83	77.6	4.42E+03	(K)YICEHQDSISGK(L)	4.4	0.6	718.822	-0.0107	-7.5	
		2	21.12	21.12	89.2	1.83E+06	(K)ADFTECCPADDK(A)	4.65	0.92	714.78	0.0121	8.5	
		3	14.78	14.78	71.6	4.00E+05	(K)ADFTECCPADDK(A)	4.57	0.99	476.8503	-0.0043	-3	
		2	18.64	18.64	82.1	2.50E+06	(R)ATYGELADCCEK(Q)	4.75	0.95	708.7914	-0.0015	-1	
		3	15.55	4.68	91.4	2.65E+05	(K)QEPERNECFLTHKDDHPNLPK(L)	4.75	1	868.7468	-0.0001	0	
		2	23.81	23.81	89.1	1.37E+07	(K)ECCHGDLLECADDR(A)	5.02	0.95	875.3341	-0.0016	-0.9	
		3	18.72	18.72	97.6	1.30E+07	(K)ECCHGDLLECADDR(A)	5.02	0.95	583.8912	-0.0035	-2	
		2	13.75	13.75	75.3	6.41E+05	(K)LCTVATLR(A)	4.93	1	467.2613	-0.0033	-3.5	
		3	22.65	13.72	100	4.16E+06	(K)LKPEPDAQCAAFQEDPDK(F)	5.2	0.92	686.9867	0.0019	0.9	
		2	24.79	17.11	93.6	7.29E+06	(K)LKPEPDAQCAAFQEDPDK(F)	5.27	0.99	1029.9761	0.0012	0.6	
		3	12.12	6.43	79.7	1.30E+06	(K)SLHTLFGDK(L)	5.67	1	339.8482	-0.0063	-6.2	
		2	19.02	9.11	92.9	1.32E+06	(K)SLHTLFGDK(L)	5.68	1	509.2697	-0.0042	-4.2	
		2	14.56	7.15	76.9	9.55E+04	(K)ADFTECCPADDKAGCLIPK(L)	6.13	0.99	1084.4755	-0.002	-0.9	
		3	13.54	13.54	85	2.16E+05	(R)RHPDYSVSLLLR(I)	6.38	0.99	485.9382	-0.0066	-4.6	

		2	20.06	20.06	92	1.44E+06	(K)LKPEPDAQCAAFQEDPDKFLGK(Y)	6.48	1	1252.61	0.0001	0	
		2	13.31	6.02	80.9	8.64E+04	(R)RHPDYSVSLLLR(I)	6.38	0.98	728.4056	-0.0027	-1.9	
		5	11.55	3.48	78.4	5.65E+05	(K)LKPEPDAQCAAFQEDPDKFLGK(Y)	6.52	0.99	501.6464	-0.0097	-3.9	
		3	13.57	13.57	78.6	4.63E+04	(R)HPDYSVSLLLR(I)	7.23	1	433.9035	-0.0096	-7.4	
		2	15.58	15.58	88.8	1.50E+06	(R)RHPYFYGPELLFHAEEYK(A)	7.57	0.99	1148.562	-0.0018	-0.8	
		3	16.4	16.4	83.2	1.12E+07	(R)HPYFYGPELLFHAEEYK(A)	8.23	0.99	714.0094	-0.0038	-1.8	
		2	14.44	4.88	84.4	2.22E+05	(K)TVLGNFSAFVAK(C)	8.13	1	627.3468	-0.0025	-2	
		2	18.3	11.08	75.4	9.28E+05	(R)HPYFYGPELLFHAEEYK(A)	8.22	0.99	1070.5112	-0.0023	-1.1	
		3	14.66	8.32	87.4	8.13E+05	(K)EDDLPSDLPALAADFAEDKEICK(H)	9.02	1	854.7339	-0.0044	-1.7	
		2	17.21	13.11	94	1.09E+04	(K)EDDLPSDLPALAADFAEDKEICK(H)	9.02	0.79	1281.5988	-0.0013	-0.5	
		2	18.87	18.87	96.7	1.34E+05	(K)EDDLPSDLPALAADFAEDK(E)	9.32	0.96	1016.4719	-0.0028	-1.4	
		3	13.66	13.66	79.8	5.85E+03	(K)EDDLPSDLPALAADFAEDK(E)	9.32	0.59	677.9821	-0.0076	-3.7	
		3	16.54	16.54	83.8	1.61E+06	(K)CCAEADPPACYATVFDQFTPLVEEPK(S)	9.5	0.96	1005.7779	-0.0018	-0.6	
		2	21.46	21.46	94.4	4.59E+05	(K)CCAEADPPACYATVFDQFTPLVEEPK(S)	9.48	0.97	1508.1608	-0.0066	-2.2	
		2	16.91	9.8	84.6	3.76E+06	(K)DVFLGTFLYEYSR(R)	9.85	0.99	805.397	-0.003	-1.8	
		3	15.07	6.64	86.7	4.44E+05	(K)DVFLGTFLYEYSR(R)	9.83	0.88	537.2648	-0.0098	-6.1	
		3	18.17	7.09	92.6	1.38E+06	(K)GLVLVAFSQYLQQCPFEDHVK(L)	9.87	1	826.7528	-0.0047	-1.9	
		4	14.34	2.68	82.3	3.30E+05	(K)GLVLVAFSQYLQQCPFEDHVK(L)	9.87	0.99	620.3161	-0.006	-2.4	
		2	13.32	6.23	76.1	1.16E+05	(K)GLVLVAFSQYLQQCPFEDHVK(L)	9.87	0.92	1239.6219	-0.0121	-4.9	
F6RI47	Uncharacterized protein	3	13.18	13.18	72.9	3.15E+05	(K)MVSGFVPLKPTVK(T)	6.33	1	468.2731	-0.0079	-5.6	2.04%
	(alpha-2-macroglobulin like)	2	15.44	15.44	85.8	9.70E+04	(K)MVSGFVPLKPTVK(T)	6.35	0.99	701.9081	-0.0037	-2.7	

		2	25.5	25.5	100	6.76E+05	(K)VVYDYYETDEFAIAEYNAPCGK(D)	7.78	0.99	1259.5375	-0.0078	-3.1	
		3	21.37	21.37	100	7.65E+05	(K)VVYDYYETDEFAIAEYNAPCGK(D)	7.8	1	840.0261	-0.0118	-4.7	
		2	12.86	5.4	77.2	9.03E+03	(K)DTIIKPLLVEPEGLEK(E)	7.87	0.72	897.5112	-0.0108	-6	
		3	14.87	9.03	91.8	8.02E+05	(K)EKFPFALEVQTLPQTCEGSK(A)	8	1	770.3861	-0.0045	-1.9	
		2	23.4	23.4	100	1.45E+05	(K)EKFPFALEVQTLPQTCEGSK(A)	8	0.98	1155.0746	-0.0063	-2.7	
		2	14.88	5.53	80.2	5.14E+04	(R)SLFTDLVAEK(D)	7.3	1	561.8026	-0.0062	-5.5	
		2	20.62	16.2	100	8.15E+04	(K)FPFALEVQTLPQTCEGSK(A)	8.45	1	1026.5085	-0.0009	-0.5	
		3	14.45	8.85	82.6	1.43E+06	(K)FPFALEVQTLPQTCEGSK(A)	8.55	1	684.6735	-0.0047	-2.3	
		3	13.11	13.11	79.8	5.70E+03	(K)DLFHCVSFIVPR(A)	8.87	0.96	497.2556	-0.0098	-6.6	
		3	19.44	19.44	89.3	6.77E+03	(K)VVYDYYETDEFAIAEYNAPCGK(D)	10.35	0.81	840.0261	-0.0118	-4.7	
F6USP9	Uncharacterized protein (Fragment)	2	20.01	11.73	86.4	3.64E+05	(R)CSTPPPSSGPTYQCLK(G)	5.05	0.98	890.4044	-0.0025	-1.4	1.24%
	(Plasminogen)	3	17.76	17.76	80.4	4.97E+05	(K)YSPDKNPSEGLEENYCR(N)	5.1	0.99	686.633	-0.0025	-1.2	
		2	22.9	22.9	100	2.93E+05	(K)YSPDKNPSEGLEENYCR(N)	5.12	0.99	1029.4448	-0.0046	-2.2	
		2	18.8	18.8	93.8	2.47E+05	(K)GPWCYTTDPGTR(F)	5.78	1	705.8079	-0.0022	-1.5	
		3	19.11	12.05	97.8	3.49E+05	(R)NPDNDEKGPWCYTTDPGTR(F)	5.88	0.99	741.6507	-0.0032	-1.4	
		2	21.9	16.83	100	1.05E+05	(R)NPDNDEKGPWCYTTDPGTR(F)	5.9	0.99	1111.9718	-0.0044	-2	
		4	15.23	7	86.7	4.05E+05	(K)TISGLECQPWASQSPHAHGYIPSK(F)	6.15	0.99	663.5707	-0.0061	-2.3	
		3	17.17	9.63	94.2	9.26E+04	(K)TISGLECQPWASQSPHAHGYIPSK(F)	6.13	0.98	884.4252	-0.006	-2.3	
		2	19.7	19.7	92.8	3.01E+05	(R)NPDGETAPWCYTTSSETR(W)	6.18	0.99	1036.4364	-0.0007	-0.3	
P07589	Fibronectin	2	12.38	1.02	81.8	1.14E+05	(R)ITGYIIK(Y)	5.48	1	404.2488	-0.0071	-8.8	1.55%
		2	15.72	8.34	95.9	7.14E+05	(R)SYTITGLQPGTDYK(I)	6.18	0.99	772.3846	-0.0019	-1.3	

		2	15.56	15.56	76.6	8.92E+04	(K)YEVSVYALK(D)	6.33	1	536.2874	-0.0046	-4.3	
		3	16.18	9.7	92.7	1.96E+05	(R)VDVIPVNLPGEHGQR(L)	6.92	0.97	543.9588	-0.0089	-5.5	
		2	19.9	8.66	90	5.62E+05	(R)TFYQIGDSWEK(Y)	7.07	0.99	687.32	-0.0045	-3.3	
		3	11.72	5.99	71.1	9.12E+04	(R)NTFAEVTGLSPGVTYHF K(V)	7.37	0.98	656.6636	-0.0099	-5	
		3	19.31	19.31	91.9	9.07E+05	(K)FTQVTPTSLTAQWTAPN VQLTGYR(V)	8.6	0.99	894.128	-0.0035	-1.3	
		2	26.62	20.4	97.8	6.36E+05	(K)FTQVTPTSLTAQWTAPN VQLTGYR(V)	8.6	0.98	1340.6893	-0.0016	-0.6	
Q28377	Fibronectin (Fragment)	2	12.38	1.02	81.8	1.14E+05	(R)ITGYIIK(Y)	5.48	1	404.2488	-0.0071	-8.8	1.69%
		2	15.72	8.34	95.9	7.14E+05	(R)SYTITGLQPGTDYK(I)	6.18	0.99	772.3846	-0.0019	-1.3	
		2	15.56	15.56	76.6	8.92E+04	(-)YEVSVYALK(D)	6.33	1	536.2874	-0.0046	-4.3	
		2	17.36	9.05	88	2.50E+06	(K)IYLYTLNDNAR(S)	6.73	0.99	678.3488	-0.0051	-3.7	
		3	17.84	10.4	86.6	1.39E+05	(R)TKTETITGFQVDAVPAN GQPPIQR(T)	6.83	0.98	856.7827	-0.0081	-3.2	
		2	23.38	17.65	98.4	4.53E+04	(K)TETITGFQVDAVPANGQ PPIQR(T)	7.17	0.98	1170.1006	-0.0051	-2.2	
		3	14.69	8.3	86.5	1.22E+04	(R)SYTITGLQPGTDYKIYLY TLNDNAR(S)	8.45	0.69	960.8155	-0.0095	-3.3	
P11276	Fibronectin	2	16.18	16.18	72.7	3.73E+04	(K)CDPIDQCQDSETR(T)	4.47	1	812.3171	-0.0104	-6.4	0.72%
		2	12.38	1.02	81.8	1.14E+05	(R)ITGYIIK(Y)	5.48	1	404.2488	-0.0071	-8.8	
		2	15.72	8.34	95.9	7.14E+05	(R)SYTITGLQPGTDYK(I)	6.18	0.99	772.3846	-0.0019	-1.3	
		2	15.56	15.56	76.6	8.92E+04	(K)YEVSVYALK(D)	6.33	1	536.2874	-0.0046	-4.3	
		2	11.76	11.76	83.1	2.06E+04	(R)TYLGNALVCTCYGGSR(G)	6.88	0.75	896.4053	-0.0119	-6.7	
		2	19.9	8.66	90	5.62E+05	(R)TFYQIGDSWEK(F)	7.07	0.99	687.32	-0.0045	-3.3	
H9GZN9	Uncharacterized protein (Fragment)	2	17.27	17.27	88.8	5.03E+05	(R)LVCQATGFSPK(E)	5	0.99	604.3086	-0.004	-3.4	1.08%
	(Immunoglobuli ln heavy chain)	2	17.9	17.9	86.3	1.45E+05	(R)DGKPVESGFTTEEVQPQ NK(E)	5.22	0.95	1045.5046	-0.0017	-0.8	

		2	11.87	11.87	79.7	1.93E+05	(K)ESWPVTYK(V)	6	1	505.251	-0.0042	-4.2	
		2	11.98	11.98	76.7	2.56E+05	(K)NFPSVLR(E)	6.18	1	416.7353	-0.0042	-5.1	
		2	12.63	1.63	80.7	2.86E+05	(K)EISVSWLR(D)	7.37	1	495.2722	-0.0043	-4.4	
		2	16.07	16.07	84.4	6.79E+04	(K)VTSMLTITESDWLNQK(V)	8.72	0.88	933.467	-0.0046	-2.5	
		3	12.25	12.25	74.8	7.01E+05	(K)TPDLFPLVSCGPSLDESLVAVGCLAR(D)	10.48	0.99	925.1328	-0.0061	-2.2	
		2	21.67	21.67	100	1.70E+05	(K)TPDLFPLVSCGPSLDESLVAVGCLAR(D)	10.47	0.97	1387.1937	-0.0098	-3.5	
P27425	Serotransferrin	2	21.9	21.9	86.2	1.36E+06	(K)AACVCQELHNQQASYGK(N)	4.7	0.98	982.4411	0	0	1.03%
		3	16.13	16.13	82.3	1.54E+04	(R)LLEACTFHR(V)	4.88	0.96	382.8608	-0.0046	-4	
		3	19.3	19.3	95.9	4.33E+05	(K)CDEWSVNSGGNIECESAQSTEDCIAK(I)	6.65	0.92	982.7287	-0.0107	-3.6	
		2	24.94	24.94	87.4	1.30E+04	(K)CDEWSVNSGGNIECESAQSTEDCIAK(I)	6.42	0.73	1473.5929	-0.0037	-1.3	
		2	14.14	7.98	85.1	1.06E+04	(K)SSSDPDLTWNSLK(G)	6.97	0.19	725.3438	-0.0053	-3.6	
		2	17.19	17.19	89.7	3.73E+05	(K)GEADAMSLDGGFIYIAGK(C)	8.68	0.98	907.934	-0.0022	-1.2	
H9GZU9	Uncharacterized protein (Fragment)	2	15	15	76.2	2.52E+05	(K)VSVTCLVK(D)	5.8	1	453.2578	-0.0041	-4.6	12.84%
	**(Immunoglobulin heavy chain)**	2	16.96	12.2	98	8.46E+04	(R)VVSVLPIQHQDWLSGK(E)	7.58	0.99	903.4975	-0.0031	-1.7	
		3	18.86	18.86	91.3	2.15E+05	(K)DFYPPEIDVEWQSNEHPEPEGK(Y)	7.75	0.88	881.3926	-0.0049	-1.9	
		2	14.06	14.06	89.5	3.52E+06	(K)APDVFPLTICGNTPDPK(V)	7.95	1	921.4582	-0.0011	-0.6	
		3	15.5	15.5	79.8	1.11E+06	(K)APDVFPLTICGNTPDPK(V)	7.93	1	614.6393	-0.0069	-3.7	
		3	22.42	22.42	100	1.57E+07	(K)VPVGCLVSNYFPEPVTVSWNCDALK(G)	9.57	0.98	951.1299	-0.0042	-1.5	
		2	21.65	21.65	97.1	5.16E+06	(K)VPVGCLVSNYFPEPVTVSWNCDALK(G)	9.57	0.97	1426.1935	0.0004	0.1	
		4	13.53	4.21	87.1	1.77E+06	(K)VPVGCLVSNYFPEPVTVSWNCDALK(G)	9.57	0.98	713.5967	-0.0144	-5	

A0A0A1E470	Immmunoglobulin lambda light chain variable region (Fragment)	2	20	20	90.8	1.77E+06	(K)VNDAVTTDGVQTTR(S)	4.52	0.99	738.8674	-0.0014	-0.9	3.22%
		2	14.89	7.46	78.3	2.45E+06	(K)YAASSYLTR(T)	4.92	1	516.2608	-0.0013	-1.3	
		2	14.72	14.72	80.1	5.99E+04	(K)VGWFQQIPGTAPK(T)	7.42	0.98	714.8834	-0.0039	-2.7	
		2	19.55	19.55	96	2.62E+06	(K)ATVVCLISDFSPSGLEVIWK(V)	10.8	1	1111.0746	-0.0154	-6.9	
F6SQD7	Uncharacterized protein (Fragment)	2	14.89	7.46	78.3	2.45E+06	(K)YAASSYLTR(T)	4.92	1	516.2608	-0.0013	-1.3	3.76%
	(Immunoglobulin light chain)	2	19.93	19.93	93.4	2.15E+06	(K)ATVVCLISDFSPSDLTVSWK(V)	10.2	1	1113.0549	-0.0133	-6	
		3	16.25	16.25	84.1	3.47E+06	(K)ATVVCLISDFSPSDLTVSWK(V)	10.2	1	742.3721	-0.0141	-6.3	
F7C0Z0	Uncharacterized protein	3	20.07	20.07	90.4	1.40E+05	(R)ECDYKDPEQAATGECTATVGR(R)	5.1	0.99	786.6708	0.0006	0.2	0.14%
	(Kininogen-1)	2	22.29	22.29	97.9	3.62E+04	(R)ECDYKDPEQAATGECTATVGR(R)	5.1	0.91	1179.5018	-0.0009	-0.4	
		2	16.47	16.47	74.5	1.03E+05	(K)YNQGNQSGYQFVLYR(I)	7.13	0.89	918.9337	-0.0062	-3.4	
		2	16.4	9.36	86.7	3.02E+04	(K)ENFLFLTPDCK(S)	8.17	0.96	692.332	-0.0046	-3.3	
P04365	Inter-alpha-trypsin inhibitor (Fragment)	2	23.65	16.63	98.4	1.53E+05	(R)TVAACNLPIVQGPCR(A)	6.32	0.99	828.4203	-0.0023	-1.4	0.11%
		2	13.64	5.62	75.1	8.16E+04	(K)CVLFTYGGCR(G)	6.32	0.99	616.78	-0.0024	-1.9	
A0A0A1E6I1	Immmunoglobulin lambda light chain variable region (Fragment)	2	15.81	15.81	86.6	1.21E+04	(R)LTIAGGPTSTPSVSLFPPSSEELSANK(A)	8.23	0.79	1344.1889	-0.0069	-2.6	2.77%
		3	15.43	15.43	83	3.04E+05	(R)LTIAGGPTSTPSVSLFPPSSEELSANK(A)	8.28	0.88	896.4627	-0.0039	-1.4	

		2	19.93	19.93	93.4	2.15E+06	(K)ATVVCLISDFSPSDLTVS WK(V)	10.2	1	1113.0549	-0.0133	-6	
		3	16.25	16.25	84.1	3.47E+06	(K)ATVVCLISDFSPSDLTVS WK(V)	10.2	1	742.3721	-0.0141	-6.3	
H9GZT5	Uncharacterized protein (Fragment)	2	19.55	19.55	92.1	2.36E+05	(K)SQTYICNVAHPASSTK(V)	4.7	0.99	882.4218	-0.0018	-1	0.23%
	(Immunoglobulin heavy chain)	2	15	15	76.2	2.52E+05	(K)VSVTCLVK(D)	5.8	1	453.2578	-0.0041	-4.6	
O97678	Transferrin (Fragment)	3	18.85	15.55	89.6	2.48E+05	(K)AVTEFESCNLAEAPNHA VVSR(K)	6.08	1	767.7001	-0.0071	-3.1	0.19%
		2	15.32	15.32	90.4	1.64E+05	(K)SGNFQLLCPDGTR(K)	6.57	0.98	732.8465	-0.0043	-2.9	
F6ZEH8	Serotransferrin	2	15.62	8.9	89.5	9.50E+04	(K)AVTEFESCNLAK(A)	5.68	1	684.8246	-0.0045	-3.3	0.12%
		2	15.32	15.32	90.4	1.64E+05	(K)SGNFQLLCPDGTR(K)	6.57	0.98	732.8465	-0.0043	-2.9	
F6W2Y1	Uncharacterized protein	2	14.77	14.77	77.1	8.26E+04	(R)DNCCILDER(F)	5.33	0.99	597.7453	-0.0045	-3.7	0.05%
	(Fibrinogen)	2	19.37	14.12	100	2.79E+04	(K)DLQDFEDILHR(A)	8.02	0.99	700.8404	-0.0069	-5	
F6RMD0	Uncharacterized protein	2	12.47	12.47	71.1	2.04E+05	(R)CYDGYTLR(G)	4.9	1	524.2302	-0.0033	-3.1	0.43%
	(Complement factor B)	2	19.23	13.49	93.4	7.23E+05	(R)LEDSVTYYCSR(G)	5.45	0.99	696.8061	-0.0051	-3.7	
F6QFD9	Uncharacterized protein	3	15.82	15.82	76	1.88E+04	(R)CTYSNPEGTVVLACDEV QCR(W)	6.8	0.96	786.6708	-0.018	-7.7	0.04%
	(Carboxypeptidase)	2	18.22	18.22	82.8	2.97E+04	(R)CTYSNPEGTVVLACDEV QCR(W)	6.78	0.9	1179.5018	-0.0196	-8.3	
		2	13.35	13.35	76.6	4.00E+04	(R)WLNIQLSPR(Q)	7.52	0.88	563.8194	-0.0052	-4.6	
													100.00%
